# Studying the impacts of variant evolution for a generalized age-group transmission model

**DOI:** 10.1371/journal.pone.0306554

**Published:** 2024-07-05

**Authors:** Fengying Wei, Ruiyang Zhou, Zhen Jin, Yamin Sun, Zhihang Peng, Shaojian Cai, Guangmin Chen, Kuicheng Zheng

**Affiliations:** 1 School of Mathematics and Statistics, Fuzhou University, Fuzhou, Fujian, China; 2 Center for Applied Mathematics of Fujian Province, Fuzhou University, Fuzhou, Fujian, China; 3 Key Laboratory of Operations Research and Control of Universities in Fujian, Fuzhou University, Fuzhou, Fujian, China; 4 Complex Systems Research Center, Shanxi University, Taiyuan, Shanxi, China; 5 Research Institute of Public Health, Nankai University, Tianjin, China; 6 Department of Biostatistics, School of Public Health, Nanjing Medical University, Nanjing, Jiangsu, China; 7 Fujian Provincial Center for Disease Control and Prevention, Fuzhou, Fujian, China; 8 Fujian Provincial Key Laboratory of Zoonosis Research, Fuzhou, Fujian, China; 9 Teaching Base of the School of Public Health of Fujian Medical University, Fuzhou, Fujian, China; Centers for Disease Control and Prevention, UNITED STATES

## Abstract

The differences of SARS-CoV-2 variants brought the changes of transmission characteristics and clinical manifestations during the prevalence of COVID-19. In order to explore the evolution mechanisms of SARS-CoV-2 variants and the impacts of variant evolution, the classic SIR (Susceptible-Infected-Recovered) compartment model was modified to a generalized SVEIR (Susceptible-Vaccinated-Exposed-Infected-Recovered) compartment model with age-group and varying variants in this study. By using of the SVEIR model and least squares method, the optimal fittings against the surveillance data from Fujian Provincial Center for Disease Control and Prevention were performed for the five epidemics of Fujian Province. The main epidemiological characteristics such as basic reproduction number, effective reproduction number, sensitivity analysis, and cross-variant scenario investigations were extensively investigated during dynamic zero-COVID policy. The study results showed that the infectivities of the variants became fast from wild strain to the Delta variant, further to the Omicron variant. Meanwhile, the cross-variant investigations showed that the average incubation periods were shortened, and that the infection scales quickly enhanced. Further, the risk estimations with the new variants were performed without implements of the non-pharmaceutical interventions, based on the dominant variants XBB.1.9.1 and EG.5. The results of the risk estimations suggested that non-pharmaceutical interventions were necessary on the Chinese mainland for controlling severe infections and deaths, and also that the regular variant monitors were still workable against the aggressive variant evolution and the emergency of new transmission risks in the future.

## Introduction

Coronavirus disease 2019 (COVID-19), caused by the severe acute respiratory syndrome coronavirus 2 (SARS-CoV-2), had raised many impacts on the global economy and public health, as reported by World Health Organization (WHO) in [1]. To fight against the spread of COVID-19 and to reduce the infection scales of severe infections and deaths, the Chinese government implemented the strict non-pharmaceutical interventions (NPIs) and nationwide vaccination on the Chinese mainland at different stages as reported in [2], during which the SARS-CoV-2 wild strain had evolved into distinct lineages as recorded in WHO [3, 4]. The transmission characteristics (transmission rates, severities, mortality rates) for the Chinese epidemics caused by the specific variants were extensively investigated in [5–11]. While, the transmission characteristics of the given Chinese province with the variant evolution were rare as of the end of 2022. According to the surveillance data from Fujian Provincial Center for Disease Control and Prevention (Fujian CDC), the five epidemics including the Fujian epidemic (Fujian Province, 2020, wild strain), the Putian epidemic (Putian City, 2021, Delta B.1.617.2), the Quanzhou epidemic (Quanzhou City, 2022, Omicron BA.2), the Xiapu epidemic (Xiapu County, 2022, Omicron BA.2.3.7), and the Fuzhou epidemic (Fuzhou City, 2022, Omicron BA.5.2) were recorded during the implementation of dynamic zero-COVID policy. The transmission characteristics of the five epidemics of Fujian Province were significant: small-scale with the clear index case, short duration with the short awareness delay, fast containment with the strict NPIs measures, which were supported by the information from the local government and the news media in [2, 12–18].

The compartment models were usually governed to describe the epidemiological and dynamic features of COVID-19 for assessing the risks and analyzing the changes of tendency. For instance, the impacts of the vaccination strategy under the multi-scenarios for the US were investigated in [19, 20]; the epidemiological differences regarding the vaccine effectiveness, the reduction of COVID-19 infection cases, the hospitalizations and deaths were explored between the US and China in [21]; the impacts of the vaccinated to the total population were concerned in [8, 11] during the implementation of dynamic zero-COVID policy by the Chinese government. Meanwhile, the recent contributions such as the media coverage and temporary immunity in [22–24]; the long-term survival analysis in [25–29]; the changes of the hospitalized/healed individuals in [30–32] and the quarantined individuals in [33, 34] discussed the dynamic behaviors and the survival analysis in detail. Under the background that the nationwide vaccination and the strict NPIs measures were kept on the Chinese mainland until the end of 2022, we established the SVEIR (Susceptible-Vaccinated-Exposed-Infected-Recovered) compartment model within the short duration of the five epidemics of Fujian Province, assumed that the changes of the vaccination for the five epidemics were not significant in this study.

The transmission mechanisms of COVID-19 were revealed that age-group played the vital roles within the total population. In detail, the differences of infection rates with age-group in [35], the hospitalization rates in [36] and the infection scales of severe infections and deaths in [8, 11, 37–40] reflected the significant differences of age-group of the total population. Meanwhile, the recent evidence regarding malaria [41], measles [42], influenza [29], and AIDS [43] revealed that age-group determined the transmission mechanisms and the further circulations. Alternatively, in the real circumstances, the policymakers of the local government and the managers of the local CDC on the Chinese mainland focused on the infection scales of severe infections and deaths for the individuals who were 60 years old and over (G2), instead of the individuals who were under 60 years old (G1), because G2 took high risks during the spread of COVID-19 due to their basic diseases (heart disease, high blood pressure, hyperlipidemia, diabetes, and chronic inflammation). Therefore, we assumed that the total population was separated into five compartments within the homogeneous mixture, and that the recovered individuals did not return into the susceptible compartment due to their temporary immunities, and further that the aging rate was a constant due to the short duration of COVID-19.

By using of the SVEIR compartment model with age-group and varying variant, the initial values of the local population and the main parameter values, the evolution features of the five epidemics of Fujian Province such as basic reproduction number, effective reproduction number, infection scales with cross-variant and potential risks of the new variants were extensively discussed in this study. With the background that the nationwide vaccination and the strict NPIs measures were implemented, the SVEIR compartment model could be applied to other epidemics with small-scale and short duration on the Chinese mainland. The main results of this study provided the potential references to the policymakers of the local government for the future risk estimations.

## Methods

### Data collection

The surveillance data ranging from January 1, 2020 to November 18, 2022 included gender, age, location, symptom onset date, diagnosis date, severity of the infection cases in this study. The surveillance data of the five epidemics of Fujian Province were granted by the Fujian CDC, which were collected from the field epidemiological investigations with the consents of all the participants, did not include any identifying information.

### A generalized age-group SVEIR model

Under the basic assumptions of this study, we denoted that the local population (i.e., *N*^*v*^(*t*)) infected by the virus *v* at the time *t* was separated into five compartments: the susceptible (i.e., *S*^*v*^(*t*)), the vaccinated (i.e., *V*^*v*^(*t*)), the exposed (i.e., *E*^*v*^(*t*)), the infected (i.e., *I*^*v*^(*t*)) and the recovered (i.e., *R*^*v*^(*t*)); further that $S_{1}^{v}\left(t\right)$, $V_{1}^{v}\left(t\right)$, $E_{1}^{v}\left(t\right)$, $I_{1}^{v}\left(t\right)$ and $R_{1}^{v}\left(t\right)$ were for G1 of the total population, $S_{2}^{v}\left(t\right)$, $V_{2}^{v}\left(t\right)$, $E_{2}^{v}\left(t\right)$, $I_{2}^{v}\left(t\right)$ and $R_{2}^{v}\left(t\right)$ were for G2 of the total population. To keep the consistency of this study in the main context and the S1 File, we let the SARS-CoV-2 wild strain for the Fujian epidemic be in purple, Delta B.1.617.2 variant for the Putian epidemic in blue, Omicron BA.2 variant for the Quanzhou epidemic in yellow, Omicron BA.2.3.7 variant for the Xiapu epidemic in brown, Omicron BA.5.2 variant for the Fuzhou epidemic in red. Moreover, all parameters of the SVEIR model were set to be constants, the descriptions of the main parameters were shown in S2 Table in S1 File. Then, the SVEIR model with age-group and varying variant was written as follows:

$$\begin{array}{c} \begin{array}{cc} \text{G1} & \left\{ \begin{array}{cc} {\dot{S}}_{1}^{v}\left(t\right) & =\Lambda^{v}-\left(\beta_{11}^{v}I_{1}^{v}+\beta_{12}^{v}I_{2}^{v}\right)\frac{S_{1}^{v}}{N^{v}}-\left(\nu_{1}^{v}+\mu_{1}^{v}+g^{v}\right)S_{1}^{v},\\ {\dot{V}}_{1}^{v}\left(t\right) & =\nu_{1}^{v}S_{1}^{v}-\left(\mu_{1}^{v}+g^{v}\right)V_{1}^{v},\\ {\dot{E}}_{1}^{v}\left(t\right) & =\left(\beta_{11}^{v}I_{1}^{v}+\beta_{12}^{v}I_{2}^{v}\right)\frac{S_{1}^{v}}{N^{v}}-\left(\alpha_{1}^{v}+\mu_{1}^{v}+g^{v}\right)E_{1}^{v},\\ {\dot{I}}_{1}^{v}\left(t\right) & =\alpha_{1}^{v}E_{1}^{v}-\left(\gamma_{1}^{v}+d_{1}^{v}+\mu_{1}^{v}+g^{v}\right)I_{1}^{v},\\ {\dot{R}}_{1}^{v}\left(t\right) & =\gamma_{1}^{v}I_{1}^{v}-\left(\mu_{1}^{v}+g^{v}\right)R_{1}^{v}, \end{array}\right.\\ \text{G2} & \left\{ \begin{array}{cc} {\dot{S}}_{2}^{v}\left(t\right) & =g^{v}S_{1}^{v}-\left(\beta_{21}^{v}I_{1}^{v}+\beta_{22}^{v}I_{2}^{v}\right)\frac{S_{2}^{v}}{N^{v}}-\left(\nu_{2}^{v}+\mu_{2}^{v}\right)S_{2}^{v},\\ {\dot{V}}_{2}^{v}\left(t\right) & =g^{v}V_{1}^{v}+\nu_{2}^{v}S_{2}^{v}-\mu_{2}^{v}V_{2}^{v},\\ {\dot{E}}_{2}^{v}\left(t\right) & =g^{v}E_{1}^{v}+\left(\beta_{21}^{v}I_{1}^{v}+\beta_{22}^{v}I_{2}^{v}\right)\frac{S_{2}^{v}}{N^{v}}-\left(\alpha_{2}^{v}+\mu_{2}^{v}\right)E_{2}^{v},\\ {\dot{I}}_{2}^{v}\left(t\right) & =g^{v}I_{1}^{v}+\alpha_{2}^{v}E_{2}^{v}-\left(\gamma_{2}^{v}+d_{2}^{v}+\mu_{2}^{v}\right)I_{2}^{v},\\ {\dot{R}}_{2}^{v}\left(t\right) & =g^{v}R_{1}^{v}+\gamma_{2}^{v}I_{2}^{v}-\mu_{2}^{v}R_{2}^{v}. \end{array}\right. \end{array} \end{array}$$

### Basic reproduction number

The basic reproduction number was defined as the average number of the secondary infections produced by an infected individual within a completely susceptible population. The expression of the basic reproduction number $R_{0}^{v}$ of the SVEIR model was provided by the next generation matrix method [44–48]. Here, the meanings of the main parameters could be found in S1, S2 Tables in S1 File.

$$\begin{array}{c} R_{0}^{v}=\frac{1}{2}(k_{11}^{v}+k_{22}^{v}+\sqrt{{\left(k_{11}^{v}\right)}^{2}-2k_{11}^{v}k_{22}^{v}+{\left(k_{22}^{v}\right)}^{2}+4k_{12}^{v}k_{21}^{v}}), \end{array}$$

with

$$\begin{array}{c} \begin{array}{cc} & k_{11}^{v}=B_{11}^{v}\frac{A_{2}^{v}C_{1}^{v}D_{2}^{v}}{A_{1}^{v}}+B_{12}^{v}(C_{2}^{v}g^{v}+\frac{A_{2}^{v}C_{1}^{v}g^{v}}{D_{1}^{v}}),\;k_{12}^{v}=B_{12}^{v}\frac{C_{2}^{v}}{A_{2}^{v}D_{2}^{v}},\\ & k_{21}^{v}=B_{21}^{v}\frac{A_{2}^{v}C_{1}^{v}D_{2}^{v}}{A_{1}^{v}}+B_{22}^{v}(C_{2}^{v}g^{v}+\frac{A_{2}^{v}C_{1}^{v}g^{v}}{D_{1}^{v}}),\;k_{22}^{v}=B_{22}^{v}\frac{C_{2}^{v}}{A_{2}^{v}D_{2}^{v}}, \end{array} \end{array}$$

and

$$\begin{array}{c} \begin{array}{cc} & A_{1}^{v}=\alpha_{1}^{v}+\mu_{1}^{v}+g^{v},\;A_{2}^{v}=\alpha_{2}^{v}+\mu_{2}^{v},\;C_{1}^{v}=\alpha_{1}^{v},\;C_{2}^{v}=\alpha_{2}^{v},\\ & B_{11}^{v}=s_{1}^{v}\left(0\right)\beta_{11}^{v},\;B_{12}^{v}=s_{1}^{v}\left(0\right)\beta_{12}^{v},\;B_{21}^{v}=s_{2}^{v}\left(0\right)\beta_{21}^{v},\;B_{22}^{v}=s_{2}^{v}\left(0\right)\beta_{22}^{v},\\ & D_{1}^{v}=\gamma_{1}^{v}+d_{1}^{v}+\mu_{1}^{v}+g^{v},\;D_{2}^{v}=\gamma_{2}^{v}+d_{2}^{v}+\mu_{2}^{v}. \end{array} \end{array}$$

### Effective reproduction number

The effective reproduction number was measured by the average number of the secondary infections by an infected individual with the time in a heterogeneously infected local population, which was a real-time indicator for the assessments of the growth or the decline of a course as reported in [49, 50]. In this study, combined with the surveillance data from Fujian CDC, the mean 4.8 and standard deviation 2.3 used in [51, 52], we adopted the EpiEstim R package in [53, 54] to produce the curves of the effective reproduction number $R_{t}^{v}$ for the five epidemics of Fujian Province. In addition, we found that the expression of the effective reproduction number with the time was well formulated in the recent contribution [55], and that the tendencies of infectious diseases such as COVID-19 in [4, 56, 57] and monkeypox in [58] were well described by using of the EpiSIX software and the surveillance data.

### Sensitivity analysis of parameters

Since the basic reproduction number $R_{0}^{v}$ was a function of the parameter *P*^*v*^, according to the sensitivity analysis in [11, 26, 40, 59], the sensitivity index *Γ* and the magnitude of impacts of $R_{0}^{v}$ with respect to the parameter *P*^*v*^ respectively were defined by

$$\begin{array}{c} \Gamma =\Gamma \left(P^{v}\right)≔\frac{\partial R_{0}^{v}}{\partial P^{v}}\cdot \frac{P^{v}}{R_{0}^{v}},\;M≔{\text{log}}_{10}\left|\Gamma \right|, \end{array}$$

where *P*^*v*^ stood for the parameter of the SVEIR model including $\beta_{11}^{v}$, $\beta_{12}^{v}$, $\beta_{21}^{v}$, $\beta_{22}^{v}$, $\gamma_{1}^{v}$, $\gamma_{2}^{v}$, $\mu_{1}^{v}$, $\mu_{2}^{v}$, $d_{1}^{v}$, $d_{2}^{v}$, $\alpha_{1}^{v}$, $\alpha_{2}^{v}$ and *g*^*v*^, also stood for the percentage of the susceptible $s_{1}^{v}\left(0\right)$ and $s_{2}^{v}\left(0\right)$, the percentage of the vaccinated $v_{1}^{v}\left(0\right)$ and $v_{2}^{v}\left(0\right)$, the proportion of aging population *p*^*v*^.

## Results

### Epidemiological characteristics of SARS-CoV-2 variants

**Fig 1:**
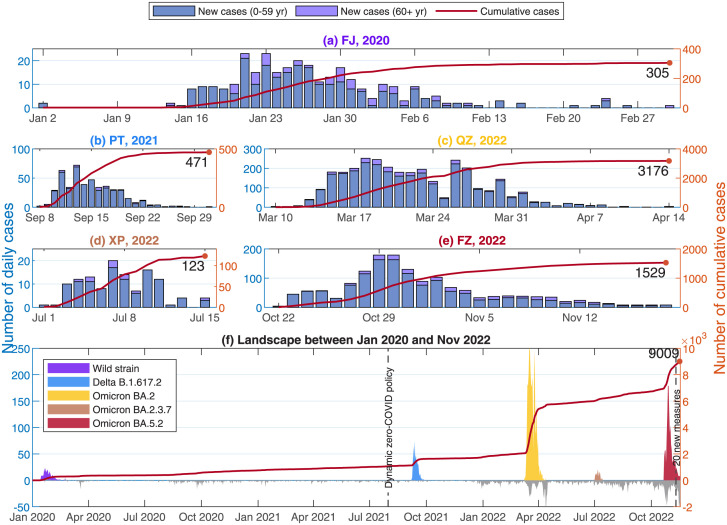
Epidemiological features and landscape between January 2020 and November 2022. Figures (a)–(e) presented the daily cases and cumulative infection cases by age-group for the five COVID-19 outbreaks and the five variants: (a) Fujian epidemic (Jan 02–Mar 01, 2020); (b) Putian epidemic (Sep 08–Oct 01, 2021); (c) Quanzhou epidemic (Mar 10–Apr 14, 2022); (d) Xiapu epidemic (Jul 01–Jul 15, 2022); (e) Fuzhou epidemic (Oct 22–Nov 18, 2022). The names for the five epidemics were matched with their corresponding variants. The red curves stood for the cumulative numbers of infection cases for the five epidemics. The blue bar meant daily new infection cases for G1, the purple bar stood for daily new infection cases for G2. Figure (f) provided landscape for the five epidemics occurred in Fujian Province from January 2020 to November 2022, in which the gray bar meant the daily new infection cases detected by closed-loop management during dynamic zero-COVID policy, referred as the infection cases from other provinces of China and other countries abroad.

**Fig 2:**
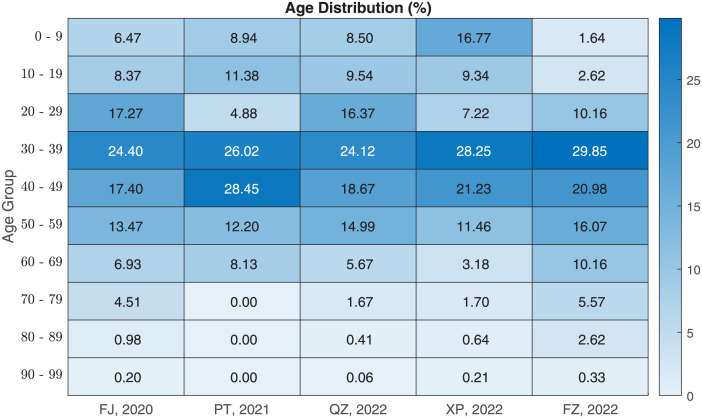
Age distribution of infection cases to the five epidemics in Fujian Province. The key population for the Fujian epidemic, the Quanzhou epidemic, the Xiapu epidemic, and the Fuzhou epidemic was 30–39 age-group, and the key population for the Putian epidemic was 40–49 age-group. School-oriented age-group included the infection cases who were under 20 years old. Job-oriented age-group clustered the infection cases whose ages ranged from 20 to 59. Home-oriented age-group collected the infection cases who were 60 years old and over. Age distribution of the Fuzhou epidemic revealed that home-oriented age-group was the key population for local governments and policymakers.

The surveillance data from Fujian CDC revealed that the first symptomatic case of COVID-19 was recorded on January 2, 2020 in the Fujian epidemic, which was led by SARS-CoV-2 wild strain with 305 infection cases as of March 1, 2020. To control the spread of COVID-19 on the Chinese mainland, dynamic zero-COVID policy was announced by the Chinese government to guarantee public health safety and economic development in August 2021 in [13]. During the implementation of dynamic zero-COVID policy, the Putian epidemic (September 8–October 1, 2021) caused by Delta B.1.617.2 was recorded with 471 infection cases. Then, the Quanzhou epidemic (March 10–April 14, 2022) caused by Omicron BA.2 was recorded with 3,176 infection cases, which was the biggest epidemic of the five epidemics. The Xiapu epidemic (July 1–July 15, 2022) caused by Omicron BA.2.3.7 was recorded with the smallest scale out of the five epidemics. The Fuzhou epidemic (October 22–November 18, 2022) caused by Omicron BA.5.2 was recorded with 1,529 infection cases, during which Twenty Measures and Ten New Measures were carried out by the Chinese government in [60, 61]. The daily infection cases and the cumulative infection cases of the five epidemics were collected by the age-group in Fig 1 and were distributed as shown in Fig 2.

### Optimal fittings of the five epidemics

**Fig 3:**
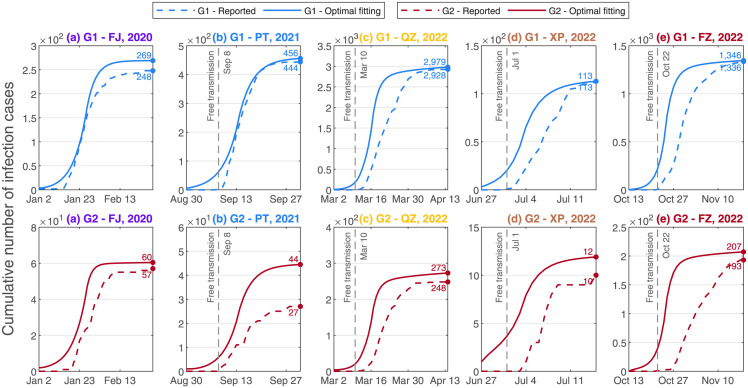
Optimal fittings by least squares method for the five epidemics in Fujian Province. Solid curves were optimal fittings, dashed curves were the surveillance data from Fujian CDC, blue curves denoted G1 of the local population, red curves meant G2 of the local population. The Pearson correlation coefficients (PCCs) of the optimal fittings were provided in S4 Table in S1 File.

The differences of the five epidemics of Fujian Province varied with the variants such as the infection scales in Fig 1, the percentages of age-group in Fig 2, and the awareness delays in S2 Table in S1 File, in which awareness delay was referred as the delay between the date of the first infection and the date of the first confirmation in [62]. By using of least squares method, the parameter values and initial values in S1, S2 Tables in S1 File, the optimal fittings against the surveillance data were performed in Fig 3. These numerical simulation results of this study were close to the recent investigations such as the awareness delay in [8, 63], the evolution trend of SARS-CoV-2 variants in [8, 36, 63, 64], the NPIs and control measures in [8, 63, 65].

### Evolution features of SARS-CoV-2 variants

**Fig 4:**
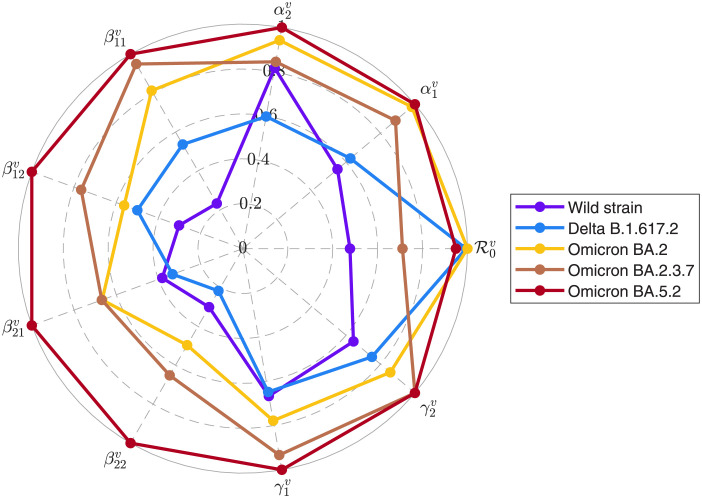
Epidemiological parameters of the five variants in Fujian Province. The values for the main epidemiological parameters were normalized between 0 and 1 by L-Infinity Norm. The colors were same with the ones of landscape in Fig 1.

A generalized SVEIR model with age-group was governed to perform the evolution features of SARS-CoV-2 variants via the optimal fittings against the surveillance data from Fujian CDC. The investigation results in Fig 4 revealed the evolution trend of key parameters. Precisely, the evolution trends of the infectious rates $\beta_{11}^{v}$, $\beta_{12}^{v}$, $\beta_{21}^{v}$ and $\beta_{22}^{v}$ were increasing when SARS-CoV-2 variants developed from the wild strain to the Omicron variant. Especially, the increasing tendencies of the infectious rates occurred within the Omicron lineage variants. This study also pointed out that, for the Omicron lineage variants, the mean incubation periods $1/\alpha_{1}^{v}$ and $1/\alpha_{2}^{v}$ increased, the mean mean inpatient periods $1/\gamma_{1}^{v}$ and $1/\gamma_{2}^{v}$ declined; and also that the values of $R_{0}^{v}$ of Omicron BA.2 and Omicron BA.5.2 clustered around that of Delta B.1.617.2.

### Scenario investigations with cross-variant

The optimal fittings of the five epidemics against the surveillance data from Fujian CDC in Fig 3 revealed the changes of SARS-CoV-2 variants, Further, the investigations of the infection scales with cross-variant presented the increasing tendencies in S2 Fig in S1 File, which reflected that the requirements of hospital-beds also showed the enhancing tendencies when SARS-CoV-2 variants developed with the time. We classified the parameters of the SVEIR model by population-oriented parameters such as Δ*T*^*v*^, Λ^*v*^, *N*^*v*^, *g*^*v*^, *p*^*v*^, $\nu_{1}^{v}$, $\nu_{2}^{v}$, $\mu_{1}^{v}$, $\mu_{2}^{v}$ and variant-oriented parameters such as $\beta_{11}^{v}$, $\beta_{12}^{v}$, $\beta_{21}^{v}$, $\beta_{22}^{v}$, $\alpha_{1}^{v}$, $\alpha_{2}^{v}$, $\gamma_{1}^{v}$, $\gamma_{2}^{v}$, $d_{1}^{v}$, $d_{2}^{v}$. When the values of population-oriented parameters were fixed, the values of variant-oriented parameters were changed in S2 Table in S1 File, the infection scales were estimated in Table 1. This study also revealed that if the Delta variant or the Omicron variant had invaded in 2020, then the infection scale would extremely exceed the surveillance data from Fujian CDC, which indicated that the infectivities of variants were greatly enhanced from the wild strain to the Delta variant, further to the Omicron variant. This study further revealed that the difficulties in prevention and control of COVID-19 were enhanced for Fujian Province during dynamic zero-COVID policy.

**Table 1:** Scenario investigations of the five epidemics with the five variants.

Variant	FJ, 2020		PT, 2021		QZ, 2022		XP, 2022		FZ, 2022	
	G1	G2	G1	G2	G1	G2	G1	G2	G1	G2
Wild strain	**269**	**60**	107	25	131	26	25	6	73	19
Delta B.1.617.2	3,615	306	**456**	**44**	625	45	48	6	208	23
Omicron BA.2	60,919	6,939	2,236	300	**2,979**	**273**	98	12	747	95
Omicron BA.2.3.7	100,702	9,976	2,914	340	4,100	326	**113**	**12**	906	101
Omicron BA.5.2	233,059	32,937	4,578	766	6,350	713	138	19	**1,346**	**207**

**Note**: FJ meant Fujian Province, PT meant Putian City, QZ meant Quanzhou City, XP meant Xiapu County, FZ meant Fuzhou City, The numbers in bold were the same with optimal fitting results in Fig 3.

### Estimation of basic reproduction number

All epidemics on the Chinese mainland were implemented NPIs despite the variant evolution kept going before the end of dynamic zero-COVID policy. Especially, with the nationwide vaccination background, the five epidemics of Fujian Province were strictly controlled by the local governments via implementing NPIs such as remote isolation, city-lockdown, centralized quarantine, contact tracing and nucleic acid testings. The infection rates in S3 Table in S1 File and other main parameters in S2 Table in S1 File were adopted to estimate the values of $R_{0}^{v}$ for the five epidemics. Thus, the estimations of $R_{0}^{v}$ for G1, G2, the total population were carried out in bold, and the changes of $R_{0}^{v}$ with cross-variant were extensively explored in Table 2.

**Table 2:** The basic reproduction numbers for the five epidemics with the five variants.

Variant	FJ, 2020			PT, 2021			QZ, 2022			XP, 2022			FZ, 2022		
	$R_{0}^{\text{G1}}$	$R_{0}^{\text{G2}}$	$R_{0}$	$R_{0}^{\text{G1}}$	$R_{0}^{\text{G2}}$	$R_{0}$	$R_{0}^{\text{G1}}$	$R_{0}^{\text{G2}}$	$R_{0}$	$R_{0}^{\text{G1}}$	$R_{0}^{\text{G2}}$	$R_{0}$	$R_{0}^{\text{G1}}$	$R_{0}^{\text{G2}}$	$R_{0}$
Wild strain	**2.42**	**0.60**	**2.55**	5.74	0.35	5.79	7.72	0.75	7.80	7.29	0.84	7.38	7.39	1.29	7.56
Delta B.1.617.2	2.21	0.67	2.39	**5.24**	**0.37**	**5.31**	7.42	0.83	7.53	6.86	0.93	6.96	7.11	1.43	7.32
Omicron BA.2	1.51	0.33	1.57	3.59	0.18	3.62	**5.31**	**0.41**	**5.35**	4.81	0.46	4.84	5.09	0.71	5.16
Omicron BA.2.3.7	0.93	0.32	0.99	2.20	0.12	2.22	4.81	0.40	4.85	**3.76**	**0.45**	**3.80**	4.61	0.69	4.68
Omicron BA.5.2	1.43	0.43	1.53	3.39	0.22	3.43	5.17	0.53	5.23	4.62	0.59	4.68	**4.95**	**0.91**	**5.07**

### Estimation of effective reproduction number

**Fig 5:**
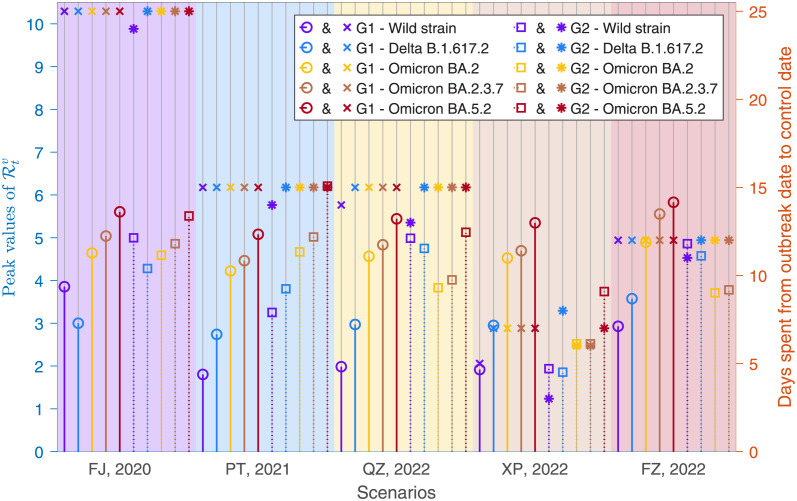
Peak values of $R_{t}^{v}$ and days spent from outbreak date to control date for the five epidemics of Fujian Province.

The effective reproduction number usually depicted the dynamic tendency for a course with the time. The dynamic tendencies $R_{t}^{v}$ of the five epidemics with cross-variant were demonstrated in S3 Fig in S1 File, by using of the Epiestim R package and the numbers of infection cases from S2 Fig in S1 File. These results showed that the maximum value of $R_{t}^{v}$ for the five epidemics was close to 6, the days spent from outbreak date to control date were about three weeks for the Fujian epidemic, two weeks for the Putian epidemic, the Quanzhou epidemic and the Fuzhou epidemic, one week for the Xiapu epidemic as presented in Fig 5.

### Sensitivity analysis of all parameters

**Fig 6:**
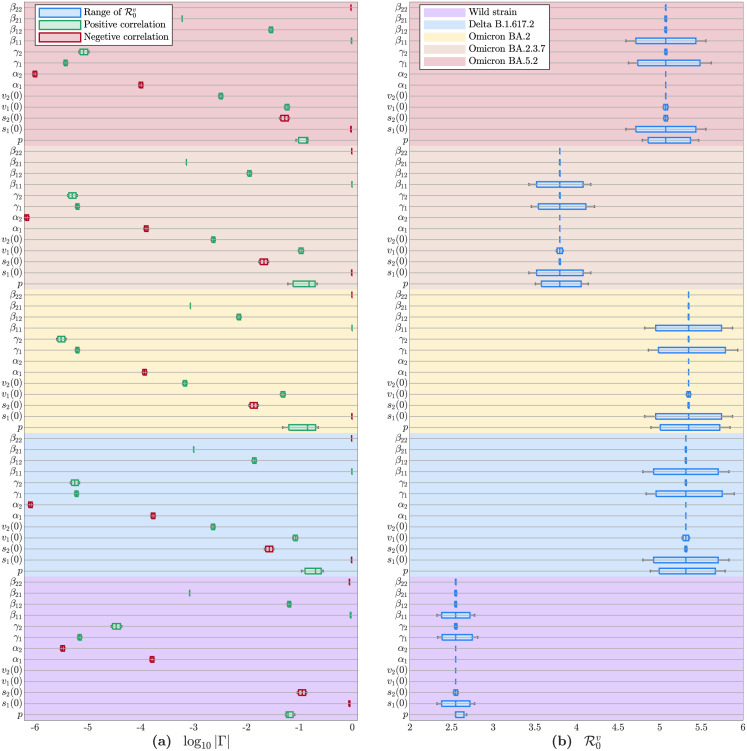
Sensitivities and variations of $R_{0}^{v}$ with respect to parameters. **(a)** The magnitude of log_10_ |Γ| indicated the sensitivity of $R_{0}^{v}$ against parameter. **(b)** Values of $R_{0}^{v}$ varied with parameters and variants.

This study showed that $\beta_{11}^{v}$, $\beta_{12}^{v}$, $\beta_{22}^{v}$, $s_{1}^{v}\left(0\right)$, $s_{2}^{v}\left(0\right)$ and *p*^*v*^ played major impacts on the transmission of the Fujian epidemic without the nationwide vaccination. While $\beta_{11}^{v}$, $\beta_{22}^{v}$, $s_{1}^{v}\left(0\right)$, $s_{2}^{v}\left(0\right)$, $v_{1}^{v}\left(0\right)$ and *p*^*v*^ played major impacts on the transmission of other four epidemics with the nationwide vaccination, as presented on the left panel of Fig 6. Especially, the increasing of $\beta_{22}^{v}$, $s_{1}^{v}\left(0\right)$ and $s_{2}^{v}\left(0\right)$ reflected the enhancements of infection scales for the five epidemics, which further implied that the implement of NPIs was beneficial to control the spread of COVID-19. This study also showed that $\beta_{11}^{v}$, $\gamma_{1}^{v}$, $s_{1}^{v}\left(0\right)$ and *p*^*v*^ were main parameters of the basic reproduction number as demonstrated on the right panel of Fig 6, of which the infection rate $\beta_{11}^{v}$ and the aging rate *p*^*v*^ were regarded as the effective indices to suppress the infection scale of COVID-19. Meanwhile, the declining average incubation periods $1/\alpha_{1}^{v}$ and $1/\alpha_{2}^{v}$ enhanced the infection scales, which further invoked more pressures on the medical runs. Consequently, the sensitivity analysis in Fig 6 showed the sensitivity of the main parameters of the SVEIR model and the important interfered insights for the epidemics on the Chinese mainland.

## Discussion

The mutation of SARS-CoV-2 variants was continuous as monitored in [4]. For instance, the XBB lineage appeared in early March 2023 on the Chinese mainland, became a dominant variant in early May 2023 (approximately 8 weeks away from the first appearance), contributed over 95.4% infection cases as of June 16 (approximately 16 weeks away from the first appearance). The latest situation report on the trend of SARS-CoV-2 variants from Chinese Center for Disease Control and Prevention showed that the proportion of the XBB lineage was close to 100% on July 30 in [66]. Among the XBB.1.9 lineages, the XBB.1.9.2 with spike mutation F456L made EG.5 in [3]. As a descendent lineage of XBB.1.9.2, the EG.5 was first reported on February 17, 2023, designated as a variant under the monitor and a variant of interest respectively on July 19 and August 9, 2023 by WHO. According to EG.5 Initial Risk Evaluation, 7,354 sequences of EG.5 had been submitted to GISAID from 51 countries, in which the largest portion of EG.5 sequences was from China (30.6%, 2,247 sequences), the EG.5 was the most reported at 49.1% from the period June 19 to July 23 compared to other XBB lineages as of August 7, 2023.

**Fig 7:**
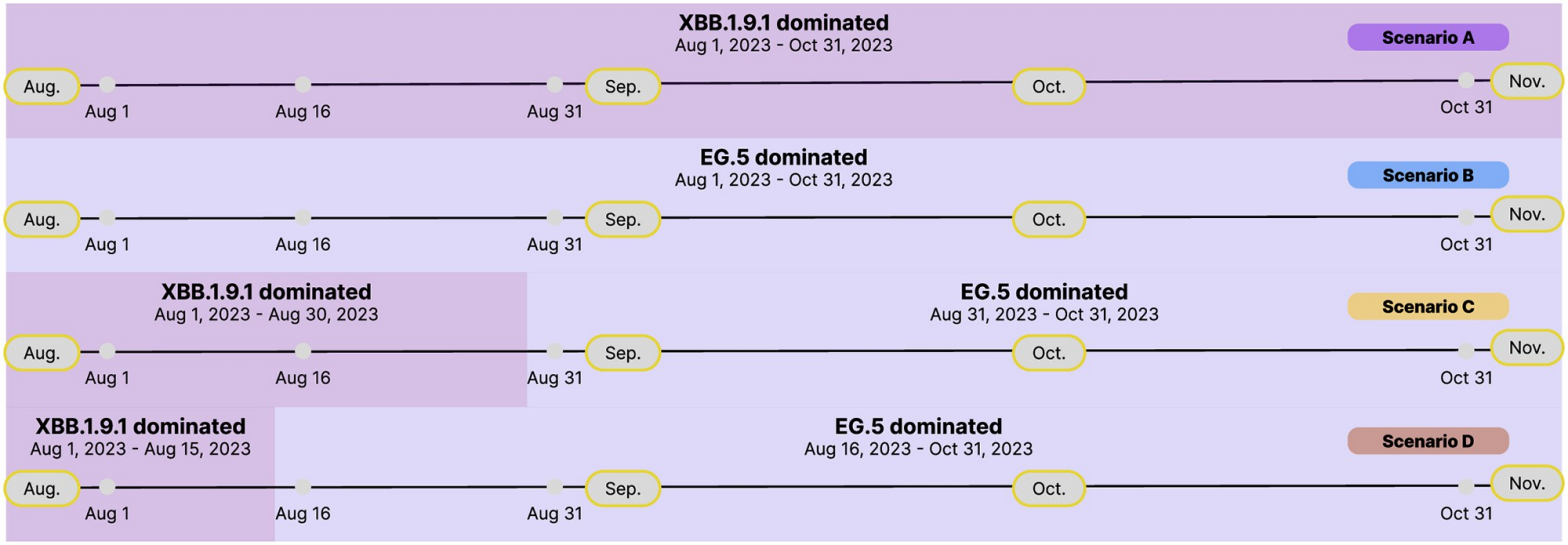
Scenario A to Scenario D. **(a)–(b)** the XBB.1.9.1 variant and the EG.5 variant dominated for three months respectively. **(c)** the XBB.1.9.1 variant dominated one month, then the EG.5 variant dominated two months. **(d)** the XBB.1.9.1 variant dominated half-month, then the EG.5 variant dominated two and a half months.

Based on the recent investigation in [4], the situation report in [66] and the appearance of the new variant EG.5 in [3], the potential COVID-19 infection scales in Fuzhou City under four scenarios were extensively investigated. Hereby, the values of the main parameters for the Fuzhou epidemic led by Omicron BA.5.2 were picked from S2, S3 Tables in S1 File, the values of the mean incubation periods and average infectious rates were reset under four scenarios. More precisely, when the XBB.1.9.1 variant dominated, the mean incubation period was set to decrease by 10%, the average infectious rates were set to increase by 10%; when the EG.5 variant dominated, the mean incubation period was set to decrease by 20%, the average infectious rates were set to increase by 20%. In more detail, the durations under four scenarios were set respectively in Fig 7. (i) Scenario A: the XBB.1.9.1 variant dominated the prevalence for three months from August 1 to October 31, 2023. (ii) Scenario B: the EG.5 variant dominated the prevalence for three months from August 1 to October 31, 2023. (iii) Scenario C: the XBB.1.9.1 variant dominated from August 1 to August 31, 2023, then the EG.5 variant dominated from September 1 to October 31, 2023. (iv) Scenario D: the XBB.1.9.1 variant dominated from August 1 to August 15, 2023, then the EG.5 variant dominated from August 16 to October 31, 2023.

**Fig 8:**
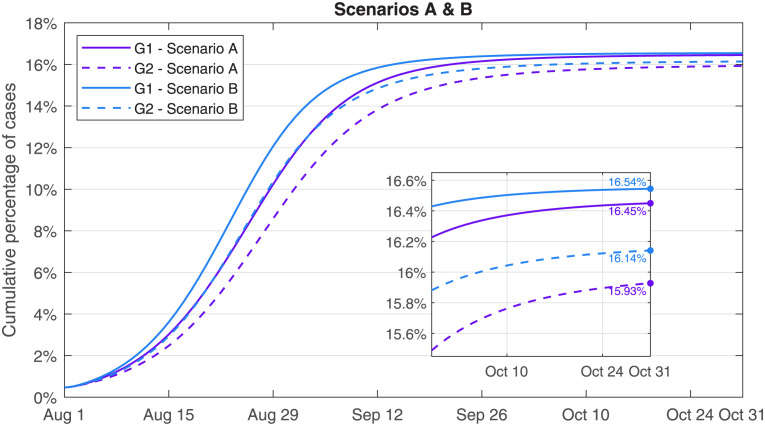
Cumulative percentage of infection cases. Purple curves were for the XBB.1.9.1 variant under Scenario A, blue curves for the EG.5 variant under Scenario B.

**Fig 9:**
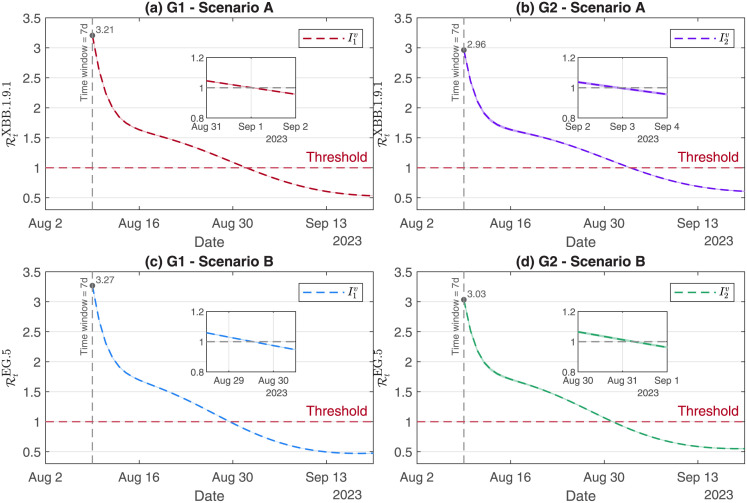
Effective reproduction numbers for G1 and G2. The dates that the epidemics were controlled for the XBB.1.9.1 variant were three days later than those for the EG.5 variant under Scenario A and Scenario B.

This study explored that, as of October 31, 2023, the cumulative percentage of infection cases to G2 under Scenario A quickly increased up to 15.93%, an extra increment of 0.21% to G2 under Scenario B was estimated in Fig 8. The values of $R_{t}^{v}$ under Scenario A were respectively controlled below one on September 1 for G1 and September 3 for G2. Compared with Scenario A, the controlled dates for $R_{t}^{v}$ were two days earlier under Scenario B in Fig 9. The numerical simulations showed that the peak was reached on September 7 with 2,635 hospital-beds under Scenario A, and that the peak was reached on September 4 with 2,868 hospital-beds under Scenario B, and also that the peak dates and infection scales under Scenario C and Scenario D were very close to the results in S4 Fig in S1 File.

### Limitations

In this study, the SVEIR model mainly focused on the epidemics of the Chinese mainland during the implementation of dynamic zero-COVID policy. The vaccination situation (January 2020-December 2022) varied from no vaccines of the country to the herd immunity with full vaccination and boosted vaccination throughout the country. While, the vaccination situation of each epidemic of Fujian Province did not change too much within a short period, according to the surveillance data from Fujian CDC. In detail, the vaccination rates from the susceptible to the vaccinated were set as the constants due to the short duration of each epidemic; the initial values of the vaccinated were supposed to be distinct values, according to the record of the surveillance data. In the real circumstances, the SVEIR model of this study brought the prompt responses against the tendency predictions of the five epidemics, which provided the potential references to the policymakers of the local government when the COVID-19 epidemic took place.

The five epidemics admitted the significant features such as small-scale and short duration due to the strict NPIs measures during the implementation of dynamic zero-COVID policy. In detail, the infection scale ranged from 123 infection cases for the Xiapu epidemic to 3,176 infection cases for the Quanzhou epidemic, the duration of the five epidemics ranged from 14 days for the Xiapu epidemic to 58 days for the Fujian epidemic, the close contacts were promptly cut by the field epidemiological investigations from Fujian CDC within two-week isolation, because the local government implemented the strict NPIs measures against the spread of COVID-19 after the index case was reported. These significant features of the five epidemics reflected the general patterns of COVID-19 on the Chinese mainland, in which each epidemic typically started with the exposure of an index case or the detection of few exposed individuals with the short awareness delay. Meanwhile, the SVEIR model with age-group and varying variant was verified to be an efficient model when the descriptions and analysis were performed for COVID-19 transmission on the Chinese mainland.

The uncertainties of the five epidemics of Fujian Province originated from the number of the index cases and the number of the close contacts. If one index case had not been included at the early field epidemiological investigations by Fujian CDC, then the infection scale caused by this index case would be enlarged with the time. If the number of the close contacts had not been clearly detected during the field epidemiological investigations, then the hidden transmission would be likely occurred in the communities until the report of symptom onset. As a consequence, the uncertainties of the epidemics on the Chinese mainland mainly came from the field epidemiological investigation and the implementation of the strict NPIs measures.

## Conclusion

A generalized SVEIR model with age-group and varying variant was built up to analyze the epidemiological features, to explore the evolution features of variants, to assess the impacts of cross-variant for the five epidemics of Fujian Province from January 2020 to November 2022. By least squares method and the surveillance data from Fujian CDC, the optimal fittings of the SVEIR model revealed that the infectivities of variants became fast before the end of dynamic zero-COVID policy. Incorporated with the variant evolution from the wild strain to the Delta variant, further to the Omicron variant, the numerical investigations with cross-variant showed that the shortened average incubation periods led to the quick increments of infection scales, which further accelerated the medical runs. The evolution assessments for the XBB.1.9.1 variant and the EG.5 variant within the next three months revealed that NPIs were necessary on the Chinese mainland for controlling severe infections and deaths, and that the regular variant monitors were still workable against the aggressive variant evolution and the emergency of new transmission risks in the future.

## Supporting information

S1 File Supplementary materials. (ZIP)
