# Supplementary material for: Studying the impacts of variant evolution for a generalized age-group transmission model
Source: PLoS One. 2024 Jul 5;19(7):e0306554. doi: 10.1371/journal.pone.0306554 (PMC11226140; doi:10.1371/journal.pone.0306554)
Supplement: S1 File — (ZIP) [file pone.0306554.s001.zip › PLoS_Supplementary_20240624.pdf]

Supplementary materials

Studying the impacts of variant evolution for a generalized age-group transmission model

Fengying Wei[a,b,c,1,*], Ruiyang Zhou[a,1,*], Zhen Jin[d,*], Yamin Sun[e], Zhihang Peng[f], Shaojian Cai[g], Guangmin Chen[g], Kuicheng Zheng[g,h,i]

[a] *School of Mathematics and Statistics, Fuzhou University, Fuzhou, 350116, Fujian, China*
[b] *Center for Applied Mathematics of Fujian Province, Fuzhou University, Fuzhou, 350116, Fujian, China*
[c] *Key Laboratory of Operations Research and Control of Universities in Fujian, Fuzhou University, Fuzhou, 350116, Fujian, China*
[d] *Complex Systems Research Center, Shanxi University, Taiyuan, 030006, Shanxi, China*
[e] *Research Institute of Public Health, Nankai University, Tianjin, 300071, China*
[f] *Department of Biostatistics, School of Public Health, Nanjing Medical University, Nanjing, 211166, Jiangsu, China*
[g] *Fujian Provincial Center for Disease Control and Prevention, Fuzhou, 350012, Fujian, China*
[h] *Fujian Provincial Key Laboratory of Zoonosis Research, Fuzhou, 350012, Fujian, China*
[i] *Teaching Base of the School of Public Health of Fujian Medical University, Fuzhou, 350012, Fujian, China*

Contents

1 Dynamics of the age-group SVEIR model **1**
1.1 A generalized age-group SVEIR model . . . 1
1.2 Disease-free equilibrium point . . . 2
1.3 Stability of disease-free equilibrium . . . 2
1.4 Endemic equilibrium point . . . 4
1.5 Stability of endemic equilibrium point . . . 7
1.6 Basic reproduction number . . . 9

2 Supplementary figures **10**
2.1 Age pyramids of the five epidemics . . . 10
2.2 Scenario comparisons . . . 11
2.3 Effective reproduction number with cross-variant . . . 13
2.4 Scenario investigations . . . 15

3 Supplementary tables **16**

1. Dynamics of the age-group SVEIR model

1.1. A generalized age-group SVEIR model

A generalized age-group SVEIR (Susceptible-Vaccinated-Exposed-Infected-Recovered) compartment model was established to investigate the dynamics of the variant evolution, the meanings of the main parameters to the SVEIR

[*] Co-corresponding authors
Email addresses: weifengying@fzu.edu.cn (Fengying Wei), ruiyangzhou@outlook.com (Ruiyang Zhou), jinzhn@263.net (Zhen Jin)
[1] These authors contributed equally to this work.

model were given in Supplementary Table 1.

$$
\mathrm{G1}\begin{cases}\dot{S}_1^v(t) = \Lambda^v - (\beta_{11}^v I_1^v + \beta_{12}^v I_2^v)\dfrac{S_1^v}{N^v} - (\nu_1^v + \mu_1^v + g^v)S_1^v,\\ \dot{V}_1^v(t) = \nu_1^v S_1^v - (\mu_1^v + g^v)V_1^v,\\ \dot{E}_1^v(t) = (\beta_{11}^v I_1^v + \beta_{12}^v I_2^v)\dfrac{S_1^v}{N^v} - (\alpha_1^v + \mu_1^v + g^v)E_1^v,\\ \dot{I}_1^v(t) = \alpha_1^v E_1^v - (\gamma_1^v + d_1^v + \mu_1^v + g^v)I_1^v,\\ \dot{R}_1^v(t) = \gamma_1^v I_1^v - (\mu_1^v + g^v)R_1^v,\end{cases}
\qquad
\mathrm{G2}\begin{cases}\dot{S}_2^v(t) = g^v S_1^v - (\beta_{21}^v I_1^v + \beta_{22}^v I_2^v)\dfrac{S_2^v}{N^v} - (\nu_2^v + \mu_2^v)S_2^v,\\ \dot{V}_2^v(t) = g^v V_1^v + \nu_2^v S_2^v - \mu_2^v V_2^v,\\ \dot{E}_2^v(t) = g^v E_1^v + (\beta_{21}^v I_1^v + \beta_{22}^v I_2^v)\dfrac{S_2^v}{N^v} - (\alpha_2^v + \mu_2^v)E_2^v,\\ \dot{I}_2^v(t) = g^v I_1^v + \alpha_2^v E_2^v - (\gamma_2^v + d_2^v + \mu_2^v)I_2^v,\\ \dot{R}_2^v(t) = g^v R_1^v + \gamma_2^v I_2^v - \mu_2^v R_2^v.\end{cases}
\tag{1}
$$

For the sake of convenience, upper case v of all variables and the parameters to model (1) were dropped throughout this Supplementary materials.

1.2. Disease-free equilibrium point

Theorem 1.1 (Existence and uniqueness of disease-free equilibrium point)**.** *For model* (1)*, disease-free equilibrium point* $X^0 = (S_1^0, S_2^0, V_1^0, V_2^0, 0, 0, 0, 0, 0, 0)^{\mathrm{T}}$ *existed, and it was unique, where* S_1^0, S_2^0, V_1^0 *and* V_2^0 *were in* (3)*.*

Proof. Let the solution of model (1) be $X = (S_1, S_2, V_1, V_2, E_1, E_2, I_1, I_2, R_1, R_2)^{\mathrm{T}}$, and the total population size be $N = S_1 + S_2 + V_1 + V_2 + E_1 + E_2 + I_1 + I_2 + R_1 + R_2$. Let $I_1 = I_2 = E_1 = E_2 = R_1 = R_2 = 0$. Model (1) then was simplified as follows:

$$
\mathrm{G1}\begin{cases}\Lambda - (\nu_1 + \mu_1 + g)S_1^0 = 0,\\ \nu_1 S_1^0 - (\mu_1 + g)\,V_1^0 = 0,\end{cases}
\qquad
\mathrm{G2}\begin{cases} gS_1^0 - (\nu_2 + \mu_2)\,S_2^0 = 0,\\ gV_1^0 + \nu_2 S_2^0 - \mu_2 V_2^0 = 0.\end{cases}
\tag{2}
$$

The equalities (2) were linear with respect to S_1^0, S_2^0, V_1^0 and V_2^0, which implied that

$$
\mathrm{G1}\begin{cases}S_1^0 = \dfrac{\Lambda}{\nu_1 + \mu_1 + g},\\ V_1^0 = \dfrac{\Lambda\nu_1}{(\mu_1 + g)(\nu_1 + \mu_1 + g)},\end{cases}
\qquad
\mathrm{G2}\begin{cases}S_2^0 = \dfrac{g}{\nu_2 + \mu_2}S_1^0,\\ V_2^0 = \dfrac{g\,(g\nu_2 + \nu_2\mu_1 + \nu_1\mu_2 + \nu_1\nu_2)}{\nu_1\mu_2(\nu_2 + \mu_2)}V_1^0.\end{cases}
\tag{3}
$$

Further, a unique disease-free equilibrium point $X^0 = (S_1^0, S_2^0, V_1^0, V_2^0, 0, 0, 0, 0, 0, 0)^{\mathrm{T}}$ was derived to model (1) with initial total population size $N^0 = S_1^0 + S_2^0 + V_1^0 + V_2^0$. □

1.3. Stability of disease-free equilibrium

Theorem 1.2 (Globally asymptotic stability of disease-free equilibrium point)**.** *If conditions* (17) *held, then disease-free equilibrium point* X^0 *was globally asymptotically stable.*

Proof. All components were re-organized and denoted by

$$
Y = (E_1, E_2, I_1, I_2, S_1, S_2, R_1, R_2, V_1, V_2), \quad Y^0 = (0, 0, 0, 0, S_1^0, S_2^0, 0, 0, V_1^0, V_2^0). \tag{4}
$$

We defined the Jacobian matrix as

$$
J = \begin{pmatrix} J_{EE} & J_{EI} & J_{ES} & J_{ER} & J_{EV}\\ J_{IE} & J_{II} & J_{IS} & J_{IR} & J_{IV}\\ J_{SE} & J_{SI} & J_{SS} & J_{SR} & J_{SV}\\ J_{RE} & J_{RI} & J_{RS} & J_{RR} & J_{RV}\\ J_{VE} & J_{VI} & J_{VS} & J_{VR} & J_{VV}\end{pmatrix}. \tag{5}
$$

Let $\eta_1 = I_1\beta_{11} + I_2\beta_{12}$, $\eta_2 = I_1\beta_{21} + I_2\beta_{22}$. Generally, the block matrices of Jacobian matrix (5) were written as follows:

$$
J_{EE} = \begin{pmatrix} -\alpha_1 - g - \mu_1 - \dfrac{S_1\eta_1}{N^2} & -\dfrac{S_1\eta_1}{N^2}\\ g - \dfrac{S_2\eta_2}{N^2} & -\alpha_2 - \mu_2 - \dfrac{S_2\eta_2}{N^2}\end{pmatrix}, \quad J_{EI} = \begin{pmatrix}\dfrac{S_1\beta_{11}}{N} - \dfrac{S_1\eta_1}{N^2} & \dfrac{S_1\beta_{12}}{N} - \dfrac{S_1\eta_1}{N^2}\\ \dfrac{S_2\beta_{21}}{N} - \dfrac{S_2\eta_2}{N^2} & \dfrac{S_2\beta_{22}}{N} - \dfrac{S_2\eta_2}{N^2}\end{pmatrix} = -J_{SI}, \tag{6}
$$

$$J_{ES} = -\eta_1 \begin{pmatrix} \dfrac{S_1}{N^2} - \dfrac{1}{N} & \dfrac{S_1}{N^2} \\ \dfrac{S_2}{N^2} & \dfrac{S_2}{N^2} - \dfrac{1}{N} \end{pmatrix}, \quad J_{ER} = J_{EV} = -\frac{1}{N^2} \begin{pmatrix} \eta_1 & \eta_1 \\ \eta_2 & \eta_2 \end{pmatrix} = J_{SR} = J_{SV}, \tag{7}$$

$$J_{IE} = \begin{pmatrix} \alpha_1 & 0 \\ 0 & \alpha_2 \end{pmatrix}, \quad J_{II} = \begin{pmatrix} -d_1 - g - \gamma_1 - \mu_1 & 0 \\ g & -d_2 - \gamma_2 - \mu_2 \end{pmatrix}, \quad J_{SE} = -\frac{1}{N^2} \begin{pmatrix} S_1\eta_1 & S_1\eta_1 \\ S_2\eta_2 & S_2\eta_2 \end{pmatrix}, \tag{8}$$

$$J_{SS} = \begin{pmatrix} \dfrac{S_1\eta_1}{N^2} - \dfrac{\eta_1}{N} - (g + \nu_1 + \mu_1) & 0 \\ g & \dfrac{S_2\eta_2}{N^2} - \dfrac{\eta_2}{N} - (\nu_2 + \mu_2) \end{pmatrix}, \tag{9}$$

$$J_{RI} = \begin{pmatrix} \gamma_1 & 0 \\ 0 & \gamma_2 \end{pmatrix}, \quad J_{RR} = \begin{pmatrix} -g - \mu_1 & 0 \\ g & -\mu_2 \end{pmatrix}, \quad J_{VS} = \begin{pmatrix} \nu_1 & 0 \\ 0 & \nu_2 \end{pmatrix}, \quad J_{VV} = \begin{pmatrix} -g - \mu_1 & 0 \\ g & -\mu_2 \end{pmatrix}. \tag{10}$$

While, the other matrices J_{IS}, J_{IR}, J_{IV}, J_{SR}, J_{RE}, J_{RS}, J_{RV}, J_{VE}, J_{VI} and J_{VR} were equal to zero.

The Jacobian matrix J at disease-free equilibrium point $X = X^0$ was

$$J_0 := J_{Y=Y^0} = \begin{pmatrix} M_1 & M_2 \\ M_3 & M_4 \end{pmatrix}, \tag{11}$$

with

$$M_1 = \begin{pmatrix} J_{EE} & J_{EI} \\ J_{IE} & J_{II} \end{pmatrix}, \quad M_2 = \begin{pmatrix} O & O & O \\ O & O & O \end{pmatrix}, \quad M_3 = \begin{pmatrix} O & J_{SI} \\ O & J_{RI} \\ O & O \end{pmatrix}, \quad M_4 = \begin{pmatrix} J_{SS} & O & O \\ O & J_{RR} & O \\ J_{VS} & O & J_{VV} \end{pmatrix}. \tag{12}$$

By the careful computation, M_1 and M_4 were respectively given as follows

$$M_1 = \begin{pmatrix} -A_1 & 0 & B_{11} & B_{12} \\ g & -A_2 & B_{21} & B_{22} \\ C_1 & 0 & -D_1 & 0 \\ 0 & C_2 & g & -D_2 \end{pmatrix}, \quad M_4 = -\begin{pmatrix} \nu_1 + \mu_1 + g & 0 & 0 & 0 & 0 & 0 \\ -g & \nu_2 + \mu_2 & 0 & 0 & 0 & 0 \\ 0 & 0 & \mu_1 + g & 0 & 0 & 0 \\ 0 & 0 & -g & \mu_2 & 0 & 0 \\ -\nu_1 & 0 & 0 & 0 & \mu_1 + g & 0 \\ 0 & -\nu_2 & 0 & 0 & -g & \mu_2 \end{pmatrix}, \tag{13}$$

where

$$\begin{aligned} &A_1 = \alpha_1 + \mu_1 + g, \quad A_2 = \alpha_2 + \mu_2, \quad C_1 = \alpha_1, \quad C_2 = \alpha_2, \\ &B_{11} = s_1(0)\beta_{11}, \quad B_{12} = s_1(0)\beta_{12}, \quad B_{21} = s_2(0)\beta_{21}, \quad B_{22} = s_2(0)\beta_{22}, \\ &D_1 = \gamma_1 + d_1 + \mu_1 + g, \quad D_2 = \gamma_2 + d_2 + \mu_2, \quad s_1(0) = S_1^0/N^0, \quad s_2(0) = S_2^0/N^0. \end{aligned} \tag{14}$$

The characteristic equation of Jacobian matrix J_0 was equal to the product of the characteristic equations of M_1 and M_4. Since the eigenvalues of M_4 were all negative, and other four eigenvalues of M_1 depended on the roots of the characteristic equation

$$\lambda^4 + L_1\lambda^3 + L_2\lambda^2 + L_3\lambda + L_4 = 0, \tag{15}$$

where

$$L_1 = A_1 + A_2 + D_1 + D_2, \tag{16a}$$

$$\begin{aligned} L_2 &= A_1A_2 + A_1D_1 + A_1D_2 + A_2D_1 + A_2D_2 - B_{11}C_1 - B_{22}C_2 + D_1D_2 \\ &= A_1A_2 + A_1D_2 + (A_1D_1 - B_{11}C_1) + (A_2D_2 - B_{22}C_2) + A_2D_1 + D_1D_2, \end{aligned} \tag{16b}$$

$$\begin{aligned} L_3 &= A_1A_2D_1 + A_1A_2D_2 - A_2B_{11}C_1 - A_1B_{22}C_2 + A_1D_1D_2 + A_2D_1D_2 - B_{11}C_1D_2 - B_{22}C_2D_1 \\ &= (A_1 + D_1)(A_2D_2 - B_{22}C_2) + (A_2 + D_2)(A_1D_1 - B_{11}C_1), \end{aligned} \tag{16c}$$

$$\begin{aligned} L_4 &= A_1A_2D_1D_2 - A_2B_{11}C_1D_2 - A_1B_{22}C_2D_1 + B_{11}B_{22}C_1C_2 - B_{12}B_{21}C_1C_2 \\ &= -A_1A_2D_1D_2 + (B_{11}B_{22} - B_{12}B_{21})C_1C_2 + A_2D_2(A_1D_1 - B_{11}C_1) + A_1D_1(A_2D_2 - B_{22}C_2). \end{aligned} \tag{16d}$$

If parameters satisfied the following inequalities:

$$A_1D_1 > B_{11}C_1, \quad A_2D_2 > B_{22}C_2, \quad (B_{11}B_{22} - B_{12}B_{21})C_1C_2 > A_1A_2D_1D_2, \tag{17}$$

then $L_1 > 0$, $L_2 > 0$, $L_3 > 0$, $L_4 > 0$. According to Routh-Hurwitz Criteria [1, 2], the additional condition for the characteristic equation (15) only admitting negative roots or negative real parts was $L_1L_2L_3 > L_3^2 + L_1^2 + L_4$. Therefore, when condition (52) and condition (53) were valid, then Theorem 1.2 held. □

1.4. Endemic equilibrium point

Let the right hand side of model (1) be zero. Then, endemic equilibrium point satisfied the following equalities.

$$\text{G1}\begin{cases} \Lambda - (\beta_{11}I_1^* + \beta_{12}I_2^*)\dfrac{S_1^*}{N^*} - (\nu_1 + \mu_1 + g)S_1^* = 0, & \text{(18a)} \\ \nu_1 S_1^* - (\mu_1 + g)\, V_1^* = 0, & \text{(18b)} \\ (\beta_{11}I_1^* + \beta_{12}I_2^*)\dfrac{S_1^*}{N^*} - (\alpha_1 + \mu_1 + g)\, E_1^* = 0, & \text{(18c)} \\ \alpha_1 E_1^* - (\gamma_1 + d_1 + \mu_1 + g)\, I_1^* = 0, & \text{(18d)} \\ \gamma_1 I_1^* - (\mu_1 + g)\, R_1^* = 0, & \text{(18e)} \end{cases}$$

$$\text{G2}\begin{cases} gS_1^* - (\beta_{21}I_1^* + \beta_{22}I_2^*)\dfrac{S_2^*}{N^*} - (\nu_2 + \mu_2)\, S_2^* = 0, & \text{(18f)} \\ gV_1^* + \nu_2 S_2^* - \mu_2 V_2^* = 0. & \text{(18g)} \\ gE_1^* + (\beta_{21}I_1^* + \beta_{22}I_2^*)\dfrac{S_2^*}{N^*} - (\alpha_2 + \mu_2)\, E_2^* = 0, & \text{(18h)} \\ gI_1^* + \alpha_2 E_2^* - (\gamma_2 + d_2 + \mu_2)\, I_2^* = 0, & \text{(18i)} \\ gR_1^* + \gamma_2 I_2^* - \mu_2 R_2^* = 0. & \text{(18j)} \end{cases}$$

Here, (18a) combined with (18c), (18f) combined with (18h), which respectively gave

$$\Lambda - (\nu_1 + \mu_1 + g)S_1^* - (\alpha_1 + \mu_1 + g)\, E_1^* = 0, \quad gS_1^* - (\nu_2 + \mu_2)\, S_2^* + gE_1^* - (\alpha_2 + \mu_2)\, E_2^* = 0, \tag{19}$$

where S_1^* and S_2^* were expressed as linear functions of E_1^* and E_2^*. Similarly, $I_1^*, I_2^*, R_1^*, R_2^*, V_1^*, V_2^*$ could also be written as linear functions of E_1^* and E_2^* by (18d)–(18g), which implied that

$$\text{G1}\begin{cases} S_1^* = \dfrac{\Lambda}{\nu_1 + \mu_1 + g} - \dfrac{\alpha_1 + \mu_1 + g}{\nu_1 + \mu_1 + g}E_1^* := u_{10} + u_{11}E_1^*, & \text{(20a)} \\ V_1^* = \dfrac{\nu_1 \Lambda}{(\mu_1 + g)(\nu_1 + \mu_1 + g)} - \dfrac{\nu_1(\alpha_1 + \mu_1 + g)}{(\mu_1 + g)(\nu_1 + \mu_1 + g)}E_1^* := y_{10} + y_{11}E_1^*, & \text{(20b)} \\ I_1^* = \dfrac{\alpha_1}{\gamma_1 + d_1 + \mu_1 + g}E_1^* := v_{11}E_1^*, & \text{(20c)} \\ R_1^* = \dfrac{\gamma_1 \alpha_1}{(\mu_1 + g)(\gamma_1 + d_1 + \mu_1 + g)}E_1^* := x_{11}E_1^*, & \text{(20d)} \end{cases}$$

$$\text{G2}\begin{cases} S_2^* = \dfrac{g\Lambda}{(\nu_1 + \mu_1 + g)(\nu_2 + \mu_2)} + \dfrac{g(\nu_1 - \alpha_1)}{(\nu_1 + \mu_1 + g)(\nu_2 + \mu_2)}E_1^* - \dfrac{\alpha_2 + \mu_2}{\nu_2 + \mu_2}E_2^* := u_{20} + u_{21}E_1^* + u_{22}E_2^*, & \text{(20e)} \\ V_2^* = \dfrac{g\nu_1 \Lambda}{\mu_2(\nu_1 + \mu_1 + g)}\left(\dfrac{1}{\mu_1 + g} + \dfrac{1}{\nu_2 + \mu_2}\right) & \\ \quad + \dfrac{g\nu_1}{\mu_2(\nu_1 + \mu_1 + g)}\left(-\dfrac{\alpha_1 + \mu_1 + g}{\mu_1 + g} + \dfrac{\nu_1 - \alpha_1}{\nu_2 + \mu_2}\right)E_1^* - \dfrac{\nu_1(\alpha_2 + \mu_2)}{\mu_2(\nu_2 + \mu_2)}E_2^* := y_{20} + y_{21}E_1^* + y_{22}E_2^*. & \text{(20f)} \\ I_2^* = \dfrac{g\alpha_1}{(\gamma_1 + d_1 + \mu_1 + g)(\gamma_2 + d_2 + \mu_2)}E_1^* + \dfrac{\alpha_2}{\gamma_2 + d_2 + \mu_2}E_2^* := v_{21}E_1^* + v_{22}E_2^*, & \text{(20g)} \\ R_2^* = \dfrac{g\alpha_1}{\mu_2(\gamma_1 + d_1 + \mu_1 + g)}\left(\dfrac{\gamma_1}{\mu_1 + g} + \dfrac{\gamma_2}{\mu_2}\right)E_1^* + \dfrac{\gamma_2\alpha_2}{\mu_2(\gamma_2 + d_2 + \mu_2)}E_2^* := x_{21}E_1^* + x_{22}E_2^*. & \text{(20h)} \end{cases}$$

The total population size N^* was computed as follows:

$$
\begin{aligned}
N^* &= S_1^* + S_2^* + E_1^* + E_2^* + I_1^* + I_2^* + R_1^* + R_2^* + V_1^* + V_2^* \\
&= u_{10} + u_{20} + y_{10} + y_{20} + (u_{11} + u_{21} + v_{11} + v_{21} + x_{11} + x_{21} + y_{11} + y_{21})E_1^* + (u_{22} + v_{22} + x_{22} + y_{22})E_2^* \qquad (21) \\
&:= w_0 + w_1E_1^* + w_2E_2^*.
\end{aligned}
$$

Together with (20) and (21), equalities (18c) and (18h) were rewritten as $f_1(E_1^*) = f_2(E_1^*) = 0$. More precisely

$$
a_{11}(E_1^*)^2 + 2a_{12}E_1^*E_2^* + 2a_1E_1^* + 2a_2E_2^* = 0, \quad b_{11}(E_1^*)^2 + 2b_{12}E_1^*E_2^* + b_{22}(E_2^*)^2 + 2b_1E_1^* + 2b_2E_2^* = 0 \qquad (22)
$$

with

$$
\begin{aligned}
& a_{11} = u_{11}\,(\beta_{11}v_{11} + \beta_{12}v_{21}) - (\alpha_1 + \mu_1 + g)\,w_1, \quad a_{12} = 2u_{11}\beta_{12}v_{21} - 2\,(\alpha_1 + \mu_1 + g)\,w_2, \\
& a_1 = 2u_{10}\,(\beta_{11}v_{11} + \beta_{12}v_{21}) - 2\,(\alpha_1 + \mu_1 + g)\,w_0, \quad a_2 = 2u_{10}\beta_{12}v_{22}, \\
& b_{11} = u_{21}\,(\beta_{21}v_{11} + \beta_{22}v_{21}) - gw_1, \quad b_{22} = u_{22}\beta_{22}v_{22} + (\alpha_2 + \mu_2)\,w_2, \\
& b_{12} = 2u_{22}\,(\beta_{21}v_{11} + \beta_{22}v_{21}) + 2u_{21}\beta_{22}v_{22} + 2\,(\alpha_2 + \mu_2)\,w_1 - 2gw_2, \\
& b_1 = 2u_{20}\,(\beta_{21}v_{11} + \beta_{22}v_{21}) - 2gw_0, \quad b_2 = 2u_{20}\beta_{22}v_{22} + 2\,(\alpha_2 + \mu_2)\,w_0.
\end{aligned} \qquad (23)
$$

The endemic equilibrium point Y^* was located at the intersection of $f_1(E_1^*) = 0$ and $f_2(E_1^*) = 0$. The careful discussions for equalities (22) were followed by cases.

Theorem 1.3 (Existence and uniqueness of the endemic equilibrium points)**.** *If one of the following conditions held, then model* (1) *had a unique endemic equilibrium point.*

(i) $a_{11} \neq 0, b_{11} \neq 0,$

$$
\begin{aligned}
& a_{11}(a_1b_{12} - a_{12}b_1)^2 = 2a_2(a_{11}b_{12} - a_{12}b_{11})(a_1b_{11} - a_{11}b_1), \\
& b_{11}(a_1b_{12} - a_{12}b_1)^2 = b_{22}(a_1b_{11} - a_{11}b_1)^2 + 2b_2(a_1b_{12} - a_{12}b_1)(a_1b_{11} - a_{11}b_1);
\end{aligned}
$$

(ii) $a_{11} = 0, b_{11} \neq 0, a_{12}b_{12} \neq 0,$

$$
\begin{aligned}
& (a_{12}b_1 + a_1b_{12} - a_2b_{11})^2 - 4a_{12}b_{12}a_1b_1 = 0, \\
& b_{12}^2(a_2b_{11} - a_1b_{12} + a_{12}b_1)^2 = [b_{11}(a_2b_{11} - a_1b_{12} - a_{12}b_1)]4a_{12}b_2b_{12} - a_1b_{12}b_{22} + a_2b_{11}b_{22} - a_{12}b_1b_{22};
\end{aligned}
$$

(iii) $a_{11} = 0, b_{11} \neq 0, a_{12}b_{12} = 0,$

$$
b_{11}(b_{22}a_1^2b_1 + 2a_2b_2b_{11}a_1 + 2a_2a_{12}b_1^2) = 2a_1b_2b_{11}(b_{12}a_1 + a_{12}b_1) + a_2^2b_1b_{11}^2 + a_{12}^2b_1^3;
$$

(iv) $a_{11} \neq 0, b_{11} = 0, a_{11}b_{22} - 2a_{12}b_{12} \neq 0,$

$$
\begin{aligned}
& (a_{12}^2b_1 - a_1a_{12}b_{12} - a_{11}a_{12}b_2 + a_1a_{11}b_{22})^2 + 2a_2a_{11}(2a_{12}b_{12} - a_{11}b_{22})(a_1b_{12} - a_{11}b_2 + a_{12}b_1) = 0, \\
& (a_{11}b_2 - a_1b_{12} - a_{12}b_1)^2 + 2a_1b_1(a_{11}b_{22} - 2a_{12}b_{12}) = 0;
\end{aligned}
$$

(v) $a_{11} \neq 0, b_{11} = 0, a_{11}b_{22} - 2a_{12}b_{12} = 0;$

(vi) $a_{11} = b_{11} = 0, a_{12}b_{22} \neq 0, (2a_{12}b_2 - 2a_2b_{12} + a_1b_{22})^2 = 8a_{12}b_{22}(a_1b_2 - a_2b_1);$

(vii) $a_{11} = b_{11} = a_{12}b_{22} = 0.$

Proof. The signs of parameters a_{11} and b_{11} in equation (22) were discussed by cases.

Case 1 ($a_{11} \neq 0$ and $b_{11} \neq 0$)

Let E_1^* be a function of E_2^*. Expression $f_1(E_1^*) = 0$ gave that

$$
E_1^* = -\frac{a_{12}E_2^* + a_1}{a_{11}} = -\frac{b_{12}E_2^* + b_1}{b_{11}} \qquad (24)
$$

when

$$
\Delta_1 = 4(a_{12}E_2^* + a_1)^2 - 8a_2a_{11}E_2^* = 0, \quad \Delta_2 = 4(b_{12}E_2^* + b_1)^2 - 4b_{11}(b_{22}E_2^* + 2b_2)E_2^* = 0. \qquad (25)
$$

Due to $f_1(E_1^*) = f_2(E_1^*) = 0$, the expressions of E_1^* and E_2^* were followed

$$
E_1^* = \frac{a_{12}b_1 - a_1b_{12}}{a_{11}b_{12} - a_{12}b_{11}}, \quad E_2^* = \frac{a_{11}b_1 - a_1b_{11}}{a_{11}b_{12} - a_{12}b_{11}}, \qquad (26)
$$

which were positive if and only if

$$(a_{12}b_1 - a_1b_{12})(a_{11}b_{12} - a_{12}b_{11}) > 0, \quad (a_{11}b_{12} - a_{12}b_{11})(a_{11}b_1 - a_1b_{11}) > 0. \tag{27}$$

Thus, condition (25) returned into the following forms.

$$\begin{aligned} &a_{11}\left(a_1b_{12} - a_{12}b_1\right)^2 = 2a_2\left(a_{11}b_{12} - a_{12}b_{11}\right)\left(a_1b_{11} - a_{11}b_1\right), \\ &b_{11}\left(a_1b_{12} - a_{12}b_1\right)^2 = b_{22}\left(a_1b_{11} - a_{11}b_1\right)^2 + 2b_2\left(a_1b_{12} - a_{12}b_1\right)\left(a_1b_{11} - a_{11}b_1\right). \end{aligned} \tag{28}$$

Case 2 ($a_{11} = 0$ and $b_{11} \neq 0$)
Equalities (22) became

$$2(a_{12}E_2^* + a_1)E_1^* + 2a_2E_2^* = 0, \quad b_{11}(E_1^*)^2 + 2(b_{12}E_2^* + b_1)E_1^* + (b_{22}E_2^* + 2b_2)E_2^* = 0, \tag{29}$$

when $\Delta_2 = (2\,b_1 + 2\,E_2^*\,b_{12})^2 - 4\,b_{11}\,(b_{22}\,E_2^{*2} + 2\,b_2\,E_2^*) = 0$, it further implied that

$$E_1^* = -\frac{b_{12}E_2^* + b_1}{b_{11}}. \tag{30}$$

Combining with the first equation of (29), which gave that

$$a_{12}b_{12}(E_2^*)^2 + (a_{12}b_1 + a_1b_{12} - a_2b_{11})E_2^* + a_1b_1 = 0. \tag{31}$$

If $a_{12}b_{12} \neq 0$ and $(a_{12}b_1 + a_1b_{12} - a_2b_{11})^2 - 4a_{12}b_{12}a_1b_1 = 0$ held, then

$$E_1^* = \frac{a_1b_{12} - a_{12}b_1 - a_2b_{11}}{2a_{12}b_{11}}, \quad E_2^* = \frac{a_2b_{11} - a_{12}b_1 - a_1b_{12}}{2a_{12}b_{12}}. \tag{32}$$

If $a_{12}b_{12} = 0$ held, then

$$E_1^* = \frac{(a_{12}b_1 - a_2b_{11})b_1}{(a_{12}b_1 + a_1b_{12} - a_2b_{11})b_{11}}, \quad E_2^* = \frac{a_1b_1}{a_2b_{11} - a_{12}b_1 - a_1b_{12}} \tag{33}$$

with

$$b_{11}(b_{22}a_1^2b_1 + 2a_2b_2b_{11}a_1 + 2a_2a_{12}b_1^2) = 2b_2b_{12}a_1^2b_{11} + 2b_2a_1a_{12}b_1b_{11} + a_2^2b_1b_{11}^2 + a_{12}^2b_1^3. \tag{34}$$

Case 3 ($a_{11} \neq 0$ and $b_{11} = 0$)
Equalities (22) turned out to be

$$a_{11}(E_1^*)^2 + 2(a_{12}E_2^* + a_1)E_1^* + 2a_2E_2^* = 0, \quad 2(b_{12}E_2^* + b_1)E_1^* + (b_{22}E_2^* + 2b_2)E_2^* = 0, \tag{35}$$

when $\Delta_1 = (2a_1 + 2E_2^*a_{12})^2 - 8E_2^*a_2a_{11} = 0$. The first equation of (35) gave that

$$E_1^* = -\frac{a_{12}E_2^* + a_1}{a_{11}}. \tag{36}$$

Combining with the second equation of (35), which yielded that

$$(a_{11}b_{22} - 2a_{12}b_{12})(E_2^*)^2 + 2(a_{11}b_2 - a_1b_{12} - a_{12}b_1)E_2^* - 2a_1b_1 = 0. \tag{37}$$

If $a_{11}b_{22} - 2a_{12}b_{12} \neq 0$ and $(a_{11}b_2 - a_1b_{12} - a_{12}b_1)^2 + 2a_1b_1(a_{11}b_{22} - 2a_{12}b_{12}) = 0$ held, then

$$E_1^* = \frac{a_{11}a_{12}b_2 + a_1a_{12}b_{12} - a_{12}^2b_1 - a_1a_{11}b_{22}}{a_{11}(a_{11}b_{22} - 2a_{12}b_{12})}, \quad E_2^* = \frac{a_{12}b_1 + a_1b_{12} - a_{11}b_2}{a_{11}b_{22} - 2a_{12}b_{12}}. \tag{38}$$

If $a_{11}b_{22} - 2a_{12}b_{12} = 0$ held, then

$$E_1^* = \frac{a_1(a_1b_{12} - a_{11}b_2)}{a_{11}(a_{11}b_2 - a_1b_{12} - a_{12}b_1)}, \quad E_2^* = \frac{a_1b_1}{a_{11}b_2 - a_1b_{12} - a_{12}b_1}. \tag{39}$$

Case 4 ($a_{11} = b_{11} = 0$)

Equalities (22) could be simplified as follows:

$$(a_{12}E_2^* + a_1)E_1^* + a_2E_2^* = 0, \quad 2(b_{12}E_2^* + b_1)E_1^* + (b_{22}E_2^* + 2b_2)E_2^* = 0. \tag{40}$$

The first equation of (40) gave that

$$E_1^* = -\frac{a_2E_2^*}{a_{12}E_2^* + a_1}, \tag{41}$$

which combined with the second equation of (40), it thus derived that

$$(a_{12}b_{22})(E_2^*)^2 + (2a_{12}b_2 - 2a_2b_{12} + a_1b_{22})E_2^* + 2(a_1b_2 - a_2b_1) = 0. \tag{42}$$

If $a_{12}b_{22} \neq 0$ and $(2a_{12}b_2 - 2a_2b_{12} + a_1b_{22})^2 = 8a_{12}b_{22}(a_1b_2 - a_2b_1)$ held, then

$$E_1^* = \frac{a_2(2a_{12}b_2 - 2a_2b_{12} + a_1b_{22})}{a_{12}(2a_2b_{12} - 2a_{12}b_2 + a_1b_{22})}, \quad E_2^* = \frac{2a_2b_{12} - 2a_{12}b_2 - a_1b_{22}}{2a_{12}b_{22}}. \tag{43}$$

If

$$a_{12}b_{22} = 0, \tag{44}$$

then

$$E_1^* = \frac{2a_2(a_1b_2 - a_2b_1)}{a_1(a_1b_{22} - 2a_2b_{12}) + 2a_{12}a_2b_1}, \quad E_2^* = -\frac{2(a_1b_2 - a_2b_1)}{2a_{12}b_2 - 2a_2b_{12} + a_1b_{22}}. \tag{45}$$

□

1.5. Stability of endemic equilibrium point

Theorem 1.4 (Globally asymptotic stability of endemic equilibrium point)**.** *Let condition* (52) *hold. Then endemic equilibrium point was globally asymptotically stable.*

Proof. We calculated the Jacobian matrix J in equation (11) at the endemic equilibrium point Y^*, and divided it into two parts as the Jacobian matrix J_a was for G1, and J_e was for G2, which were respectively presented as follows:

$$J_a = \begin{pmatrix} -\alpha_1 - g - \mu_1 - \dfrac{S_1^*\eta_1}{(N^*)^2} & \dfrac{S_1^*\beta_{11}}{N^*} - \dfrac{S_1^*\eta_1}{(N^*)^2} & -\eta_1\left(\dfrac{S_1^*}{(N^*)^2} - \dfrac{1}{N^*}\right) & -\dfrac{\eta_1}{(N^*)^2} & -\dfrac{\eta_1}{(N^*)^2} \\ \alpha_1 & -d_1 - g - \gamma_1 - \mu_1 & 0 & 0 & 0 \\ -\dfrac{S_1^*\eta_1}{(N^*)^2} & \dfrac{S_1^*\eta_1}{(N^*)^2} - \dfrac{S_1^*\beta_{11}}{N^*} & \eta_1\left(\dfrac{S_1^*}{(N^*)^2} - \dfrac{1}{N^*}\right) - g - \nu_1 - \mu_1 & \dfrac{\eta_1}{(N^*)^2} & \dfrac{\eta_1}{(N^*)^2} \\ 0 & \gamma_1 & 0 & -g - \mu_1 & 0 \\ 0 & 0 & \nu_1 & 0 & -g - \mu_1 \end{pmatrix}, \tag{46}$$

$$J_e = \begin{pmatrix} -\alpha_2 - \mu_2 - \dfrac{S_2^*\eta_2}{(N^*)^2} & \dfrac{S_2^*\beta_{22}}{N^*} - \dfrac{S_2^*\eta_2}{(N^*)^2} & -\eta_2\left(\dfrac{S_2^*}{(N^*)^2} - \dfrac{1}{N^*}\right) & -\dfrac{\eta_2}{(N^*)^2} & -\dfrac{\eta_2}{(N^*)^2} \\ \alpha_2 & -d_2 - \gamma_2 - \mu_2 & 0 & 0 & 0 \\ \dfrac{S_2^*\eta_2}{(N^*)^2} & \dfrac{S_2^*\eta_2}{(N^*)^2} - \dfrac{S_2^*\beta_{22}}{N^*} & \eta_2\left(\dfrac{S_2^*}{(N^*)^2} - \dfrac{1}{N^*}\right) - \nu_2 - \mu_2 & \dfrac{\eta_2}{(N^*)^2} & \dfrac{\eta_2}{(N^*)^2} \\ 0 & \gamma_2 & 0 & -\mu_2 & 0 \\ 0 & 0 & \nu_2 & 0 & -\mu_2 \end{pmatrix}. \tag{47}$$

Since the signs at $(i,\ j)$-element for both J_a and J_e were the same for $i,\ j = 1,\ 2,\ 3,\ 4,\ 5$, which was denoted by

$$M = \begin{pmatrix} -m_{11} & m_{12} & m_{13} & -m_{14} & -m_{14} \\ m_{21} & -m_{22} & 0 & 0 & 0 \\ -m_{31} & -m_{12} & -m_{33} & m_{14} & m_{14} \\ 0 & m_{42} & 0 & -m_{44} & 0 \\ 0 & 0 & m_{53} & 0 & -m_{44} \end{pmatrix}. \tag{48}$$

Taking $M = J_e$, the corresponding characteristic equation of M was computed as follows:

$$\lambda^5 + L_1\lambda^4 + L_2\lambda^3 + L_3\lambda^2 + L_4\lambda + L_5 = 0, \tag{49}$$

with its coefficients

$$L_1 = m_{11} + m_{22} + m_{33} + 2m_{44} > 0, \tag{50a}$$

$$\begin{aligned} L_2 &= m_{11}m_{22} - m_{12}m_{21} + m_{11}m_{33} + m_{13}m_{31} + 2m_{11}m_{44} + m_{22}m_{33} \\ &\quad - m_{14}m_{53} + 2m_{22}m_{44} + 2m_{33}m_{44} + m_{44}^2 \\ &= (m_{11}m_{22} - m_{12}m_{21}) + m_{11}m_{33} + (m_{13}m_{31} - m_{14}m_{53}) \\ &\quad + 2m_{11}m_{44} + m_{22}m_{33} + 2m_{22}m_{44} + 2m_{33}m_{44} + m_{44}^2, \end{aligned} \tag{50b}$$

$$\begin{aligned} L_3 &= m_{11}m_{44}^2 - m_{11}m_{14}m_{53} + m_{22}m_{44}^2 + m_{33}m_{44}^2 + m_{12}m_{13}m_{21} + m_{11}m_{22}m_{33} \\ &\quad - m_{12}m_{21}m_{33} + m_{13}m_{22}m_{31} + m_{11}m_{21}m_{42} + 2m_{11}m_{22}m_{44} - 2m_{12}m_{21}m_{44} - m_{14}m_{22}m_{53} \\ &\quad + 2m_{11}m_{33}m_{44} + 2m_{13}m_{31}m_{44} - m_{11}m_{31}m_{53} + 2m_{22}m_{33}m_{44} - m_{11}m_{44}m_{53} \\ &= m_{11}m_{44}^2 + m_{11}(m_{33}m_{44} - m_{11}m_{53}) + m_{22}m_{44}^2 + m_{33}m_{44}^2 + m_{12}m_{13}m_{21} \\ &\quad + m_{33}(m_{11}m_{22} - m_{12}m_{21}) + m_{22}(m_{13}m_{31} - m_{14}m_{53}) + m_{11}m_{21}m_{42} + 2m_{44}(m_{11}m_{22} - m_{12}m_{21}) \\ &\quad + m_{11}m_{33}m_{44} + m_{31}(m_{13}m_{44} - m_{14}m_{53}) + 2m_{22}m_{33}m_{44} + m_{44}(m_{13}m_{31} - m_{14}m_{53}), \end{aligned} \tag{50c}$$

$$\begin{aligned} L_4 &= m_{11}m_{22}m_{44}^2 - m_{12}m_{21}m_{44}^2 - m_{11}m_{14}m_{22}m_{53} + m_{11}m_{33}m_{44}^2 + m_{13}m_{31}m_{44}^2 + m_{22}m_{33}m_{44}^2 \\ &\quad - m_{11}m_{14}m_{44}m_{53} - m_{11}m_{13}m_{21}m_{42} + 2m_{12}m_{13}m_{21}m_{44} + m_{11}m_{21}m_{33}m_{42} \\ &\quad + 2m_{11}m_{22}m_{33}m_{44} - 2m_{12}m_{21}m_{33}m_{44} + 2m_{13}m_{22}m_{31}m_{44} - m_{11}m_{22}m_{31}m_{53} \\ &\quad + m_{11}m_{21}m_{42}m_{44} - m_{11}m_{22}m_{44}m_{53} - m_{11}m_{31}m_{44}m_{53} \\ &= m_{44}^2(m_{11}m_{22} - m_{12}m_{21}) + m_{12}m_{13}m_{21}m_{44} - m_{11}m_{14}m_{22}m_{53} + m_{11}m_{44}(m_{33}m_{44} - m_{11}m_{53}) \\ &\quad + m_{31}m_{44}(m_{13}m_{44} - m_{14}m_{53}) + m_{22}m_{33}m_{44}^2 + m_{12}m_{13}m_{21}m_{44} + m_{11}m_{21}(m_{33}m_{42} - m_{13}m_{42}) \\ &\quad + 2m_{33}m_{44}(m_{11}m_{22} - m_{12}m_{21}) + m_{22}m_{31}(m_{13}m_{44} - m_{14}m_{53}) \\ &\quad + m_{11}m_{21}m_{42}m_{44} + m_{22}m_{44}(m_{13}m_{31} - m_{14}m_{53}), \end{aligned} \tag{50d}$$

$$\begin{aligned} L_5 &= m_{12}m_{13}m_{21}m_{44}^2 + m_{11}m_{22}m_{33}m_{44}^2 - m_{12}m_{21}m_{33}m_{44}^2 + m_{13}m_{22}m_{31}m_{44}^2 \\ &\quad - m_{11}m_{14}m_{22}m_{44}m_{53} - m_{13}m_{14}m_{21}m_{42}m_{44} + m_{14}m_{21}m_{33}m_{42}m_{44} - m_{14}m_{22}m_{31}m_{44}m_{53} \\ &= m_{44}(m_{12}m_{13}m_{21}m_{44} - m_{11}m_{14}m_{22}m_{53}) \\ &= m_{33}m_{44}^2(m_{11}m_{22} - m_{12}m_{21}) + m_{22}m_{31}m_{44}(m_{13}m_{44} - m_{14}m_{53}) + m_{14}m_{21}m_{42}m_{44}(m_{33} - m_{13}). \end{aligned} \tag{50e}$$

If the parameters satisfied the following inequalities:

$$\begin{aligned} &m_{12}m_{13}m_{21}m_{44} - m_{11}m_{14}m_{22}m_{53} > 0, \quad m_{33} - m_{13} > 0, \\ &m_{11}m_{22} - m_{12}m_{21} > 0, \quad m_{13}m_{44} - m_{14}m_{53} > 0, \quad m_{33}m_{44} - m_{11}m_{53} > 0, \end{aligned} \tag{51}$$

then $L_1 > 0$, $L_2 > 0$, $L_3 > 0$, $L_4 > 0$, $L_5 > 0$. Due to $m_{33} - m_{13} = \nu_2 + \mu_2 > 0$ in (48), then condition (51) was simplified as

$$\begin{aligned} &m_{12}m_{13}m_{21}m_{44} - m_{11}m_{14}m_{22}m_{53} > 0, \quad m_{11}m_{22} - m_{12}m_{21} > 0, \\ &m_{13}m_{44} - m_{14}m_{53} > 0, \quad m_{33}m_{44} - m_{11}m_{53} > 0. \end{aligned} \tag{52}$$

According to Routh-Hurwitz Criteria [1, 2], the additional condition that guaranteed the characteristic equation (49) admitting negative roots or negative real parts was

$$L_1L_2L_3 > L_3^2 + L_1^2L_4, \quad (L_1L_4 - L_5)(L_1L_2L_3 - L_3^2 - L_1^2L_4) > L_5(L_1L_2 - L_3)^2 + L_1L_5^2. \tag{53}$$

Therefore, when conditions (52) and (53) held, the endemic equilibrium point was globally asymptotically stable. □

1.6. Basic reproduction number

The basic production number $\mathcal{R}_0$ described the number of secondary infections produced by a primary infection in a completely susceptible population. The expression of the basic reproduction number $\mathcal{R}_0$ was computed by using the next generation matrix method given in [3]. The matrix of the new infective individuals in compartments I and E of model (1) was firstly written as:

$$\mathcal{F} = \Big((\beta_{11}I_1 + \beta_{12}I_2)\frac{S_1}{N},\ (\beta_{21}I_1 + \beta_{22}I_2)\frac{S_2}{N},\ 0,\ 0,\ \cdots,\ 0\Big)^{\mathrm{T}}. \tag{54}$$

Let the matrix except for the new infective individuals of model (1) be $\mathcal{V}$. The components were respectively

$$\mathcal{V} = \mathcal{V}^- - \mathcal{V}^+ = (Q_1,\ Q_2,\ Q_3,\ Q_4,\ Q_5)^{\mathrm{T}} - (P_1,\ P_2,\ P_3,\ P_4,\ P_5)^{\mathrm{T}}, \tag{55}$$

here, $\mathcal{V}^-$ stood for the transfer matrix of the individuals from compartment I, and $\mathcal{V}^+$ stood for the transfer matrix of the individuals into compartment I with

$$\begin{aligned} &P_1 = ((\alpha_1 + \mu_1 + g)E_1,\ (\alpha_2 + \mu_2)E_2), \quad P_2 = ((\gamma_1 + d_1 + \mu_1 + g)I_1,\ (\gamma_2 + d_2 + \mu_2)I_2), \\ &P_3 = ((\nu_1 + \mu_1 + g)S_1,\ (\nu_2 + \mu_2)S_2), \quad P_4 = ((\mu_1 + g)R_1,\ \mu_2 R_2), \quad P_5 = ((\mu_1 + g)V_1,\ \mu_2 V_2), \end{aligned} \tag{56a}$$

$$\begin{aligned} &Q_1 = (0,\ gE_1), \quad Q_2 = (\alpha_1 E_1,\ gI_1 + \alpha_2 E_2), \\ &Q_3 = (\Lambda,\ gS_1), \quad Q_4 = (\gamma_1 I_1,\ gR_1 + \gamma_2 I_2), \quad Q_5 = (\nu_1 S_1,\ gV_1 + \nu_2 S_2). \end{aligned} \tag{56b}$$

Next, the first four components of $\mathcal{F}$ and $\mathcal{V}$ were picked to compute F and V by Lemma 1 in [3], for $i,\ j = 1,\ 2,\ 3,\ 4$, which gave that

$$F = \begin{pmatrix} 0 & 0 & B_{11} & B_{12} \\ 0 & 0 & B_{21} & B_{22} \\ 0 & 0 & 0 & 0 \\ 0 & 0 & 0 & 0 \end{pmatrix},\ V = \begin{pmatrix} A_1 & 0 & 0 & 0 \\ -g & A_2 & 0 & 0 \\ -C_1 & 0 & D_1 & 0 \\ 0 & -C_2 & -g & D_2 \end{pmatrix},\ \text{and } FV^{-1} := \begin{pmatrix} k_{11} & k_{12} & k_{13} & k_{14} \\ k_{21} & k_{22} & k_{23} & k_{24} \\ 0 & 0 & 0 & 0 \\ 0 & 0 & 0 & 0 \end{pmatrix}, \tag{57}$$

with

$$\begin{aligned} &k_{11} = B_{11}\frac{A_2C_1D_2}{A_1} + B_{12}g\left(C_2 + \frac{A_2C_1}{D_1}\right), \quad k_{21} = B_{21}\frac{A_2C_1D_2}{A_1} + B_{22}g\left(C_2 + \frac{A_2C_1}{D_1}\right), \\ &k_{12} = B_{12}\frac{C_2}{A_2D_2}, \quad k_{22} = B_{22}\frac{C_2}{A_2D_2}, \quad k_{13} = B_{11}\frac{D_2}{D_1} + B_{12}\frac{g}{D_1}, \quad k_{23} = B_{21}\frac{d_2 + \mu_2}{D_1} + B_{22}\frac{g + \gamma_2}{D_1}, \\ &k_{14} = \frac{B_{12}}{D_2}, \quad k_{24} = \frac{B_{22}}{D_2}, \quad B_{11} = s_1(0)\beta_{11}, \quad B_{12} = s_1(0)\beta_{12}, \quad B_{21} = s_2(0)\beta_{21}, \quad B_{22} = s_2(0)\beta_{22}. \end{aligned} \tag{58}$$

Here $k_{ij} > 0$ were assumed to be positive with $i = 1,\ 2,\ j = 1,\ 2,\ 3,\ 4$. The characteristic equation of matrix FV^{-1} was written as $\lambda^2\left[(\lambda - k_{11})(\lambda - k_{22}) - k_{12}k_{21}\right] = 0$, all eigenvalues were computed as $\lambda_1 = 0$, $\lambda_2 = 0$ and

$$\begin{aligned} \lambda_3 &= \frac{1}{2}\left(k_{11} + k_{22} - \sqrt{k_{11}^2 - 2k_{11}k_{22} + k_{22}^2 + 4k_{12}k_{21}}\right), \\ \lambda_4 &= \frac{1}{2}\left(k_{11} + k_{22} + \sqrt{k_{11}^2 - 2k_{11}k_{22} + k_{22}^2 + 4k_{12}k_{21}}\right), \end{aligned} \tag{59}$$

with $k_{11}^2 - 2k_{11}k_{22} + k_{22}^2 + 4k_{12}k_{21} = (k_{11} - k_{22})^2 + 4k_{12}k_{21} > 0$. So, the basic reproduction number $\mathcal{R}_0$ (also referred as the spectral radius of matrix FV^{-1}) was given by the following expression:

$$\mathcal{R}_0 = \rho(FV^{-1}) = \max\{\lambda : \|\lambda\mathbb{I}_4 - FV^{-1}\| = 0\} = \frac{1}{2}\left(k_{11} + k_{22} + \sqrt{k_{11}^2 - 2k_{11}k_{22} + k_{22}^2 + 4k_{12}k_{21}}\right), \tag{60}$$

where $\mathbb{I}_4$ was an identity matrix of type $4{\times}4$, k_{ij} depended on age-structure of the local population for $i,\ j = 1,\ 2,\ 3,\ 4$.

According to Theorem 2 in [3], the following corollary was derived.

Corollary 1.5 (Locally asymptotic stability of disease-free equilibrium point). *If $\mathcal{R}_0 < 1$, then disease-free equilibrium X^0 was locally asymptotically stable; otherwise, X^0 was unstable.*

2. Supplementary figures

2.1. *Age pyramids of the five epidemics*

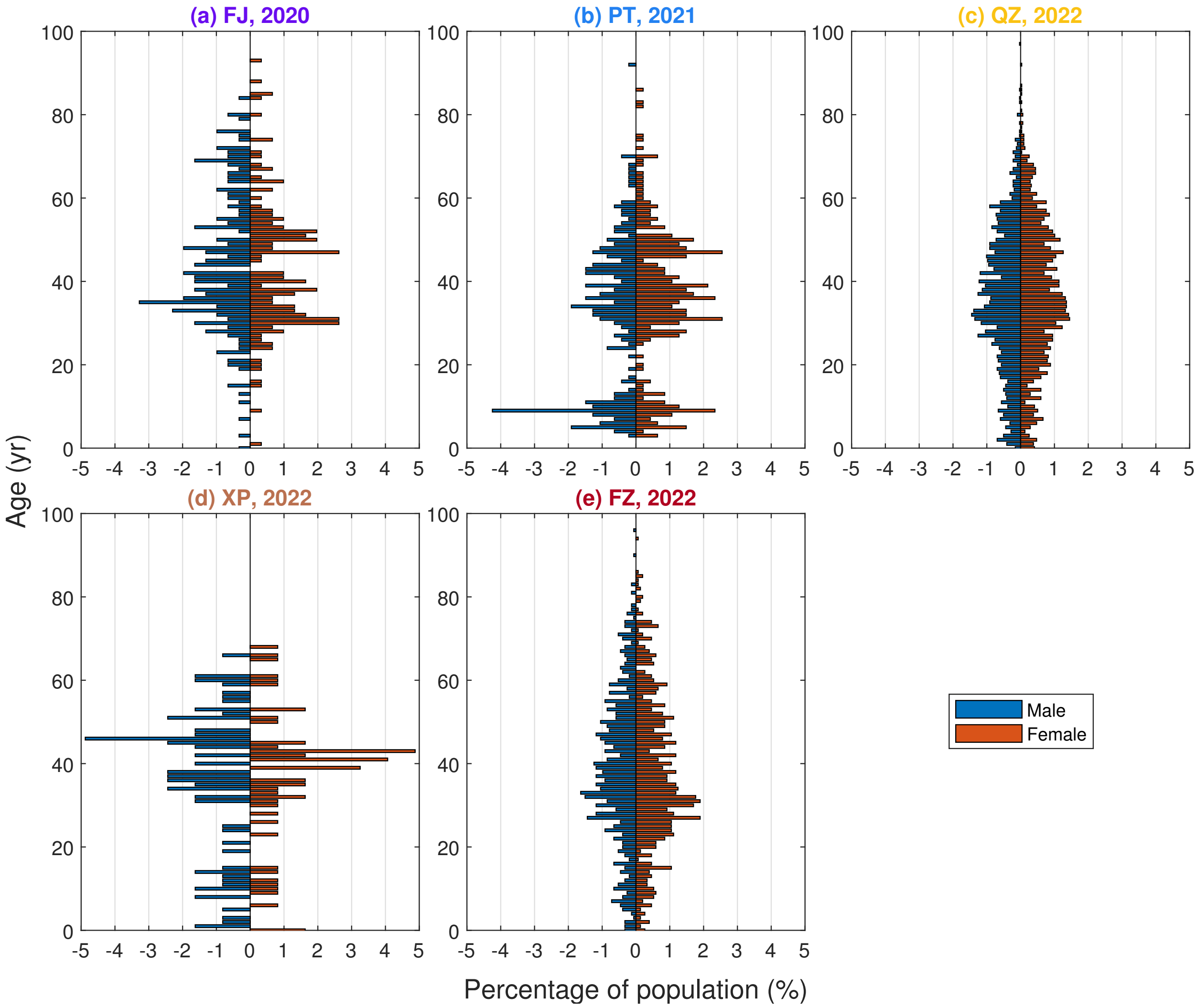

Supplementary Fig 1. Age pyramids of the five epidemics. (a) Fujian epidemic (Jan 02–Mar 01); (b) Putian epidemic (Sep 08–Oct 01); (c) Quanzhou epidemic (Mar 10–Apr 14); (d) Xiapu epidemic (Jul 01–Jul 15); (e) Fuzhou epidemic (Oct 22–Nov 18). Blue bar meant the percentage of the male, red bar meant the percentage of the female.

2.2. Scenario comparisons

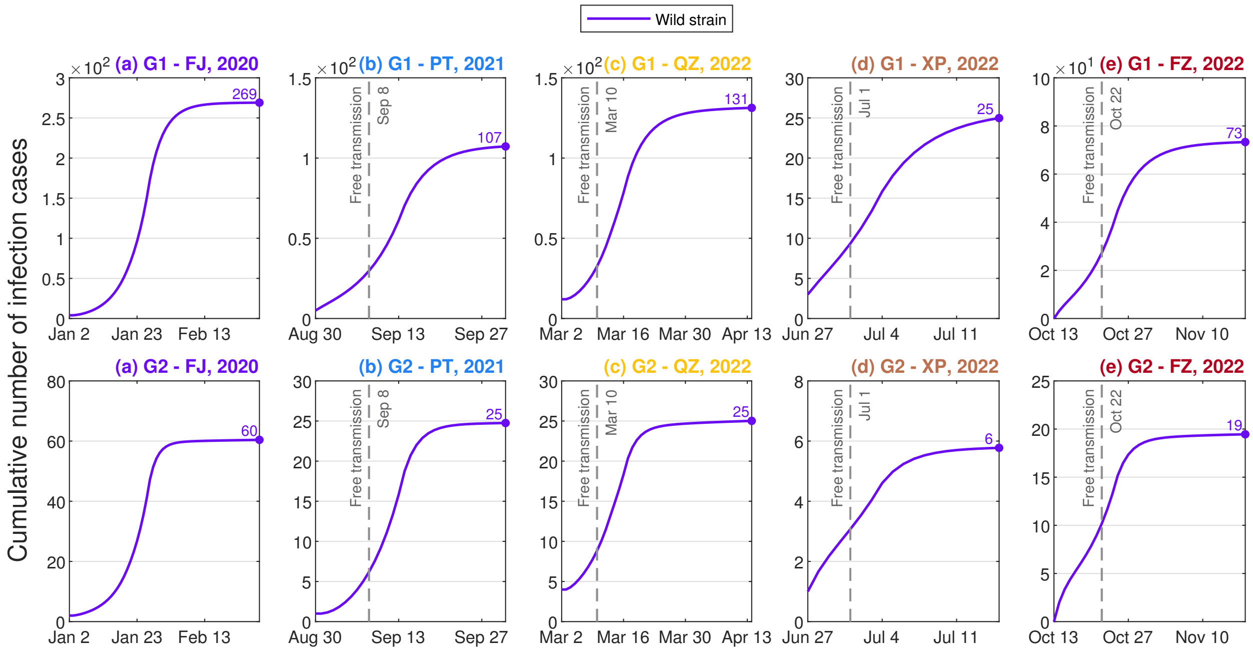

(a) The five epidemics under Wild strain.

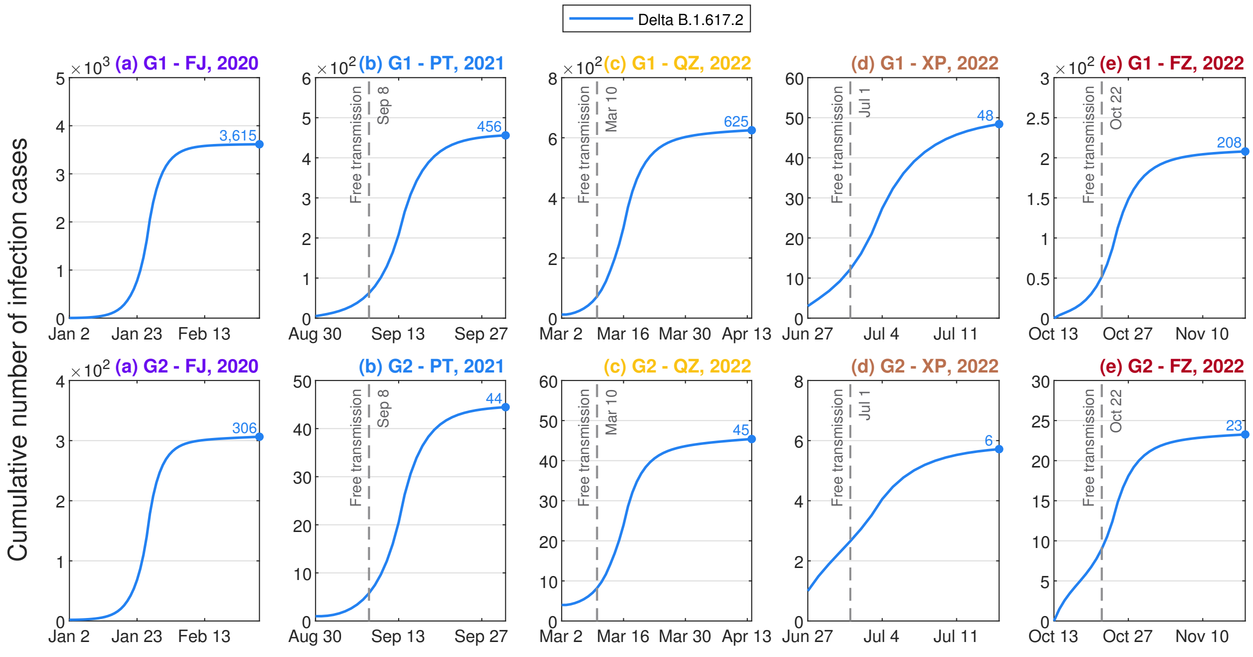

(b) The five epidemics under Delta B.1.617.2.

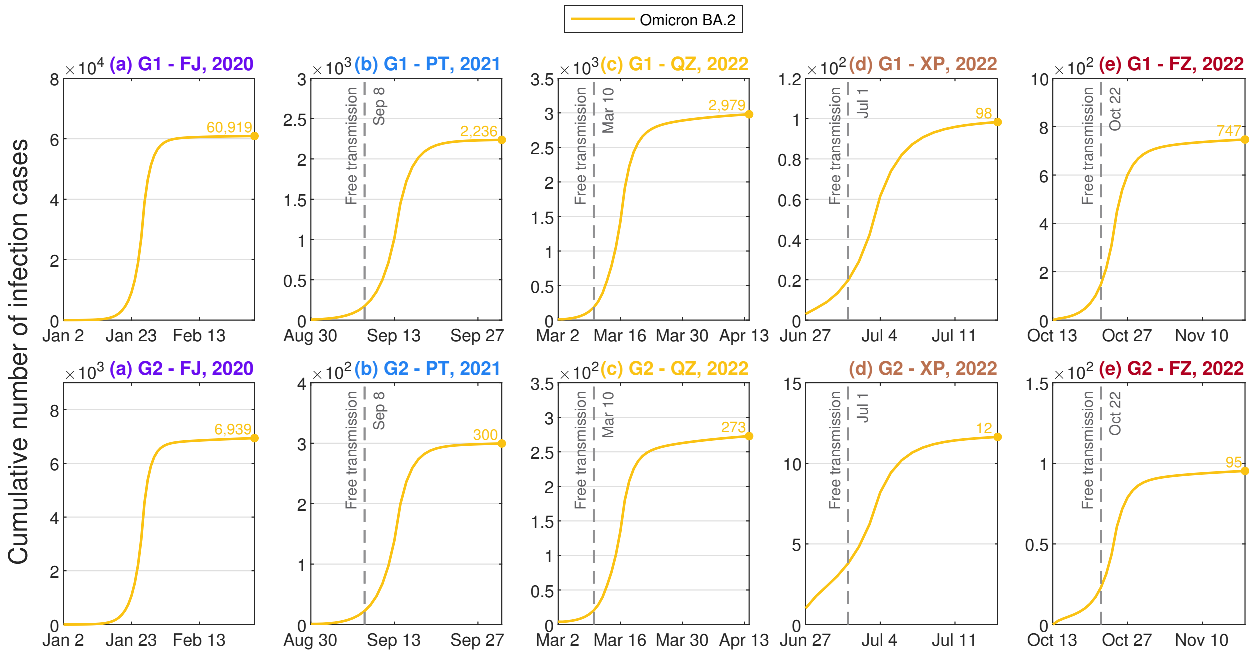

(c) The five epidemics under Omicron BA.2.

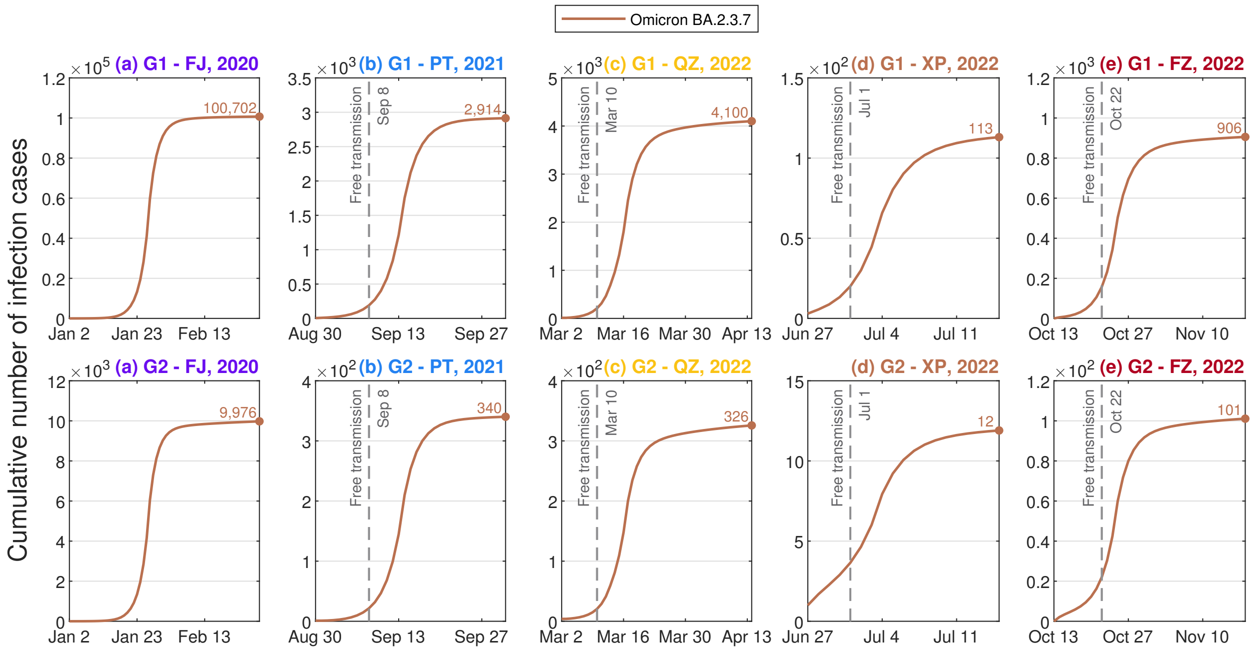

(d) The five epidemics under Omicron BA.2.3.7.

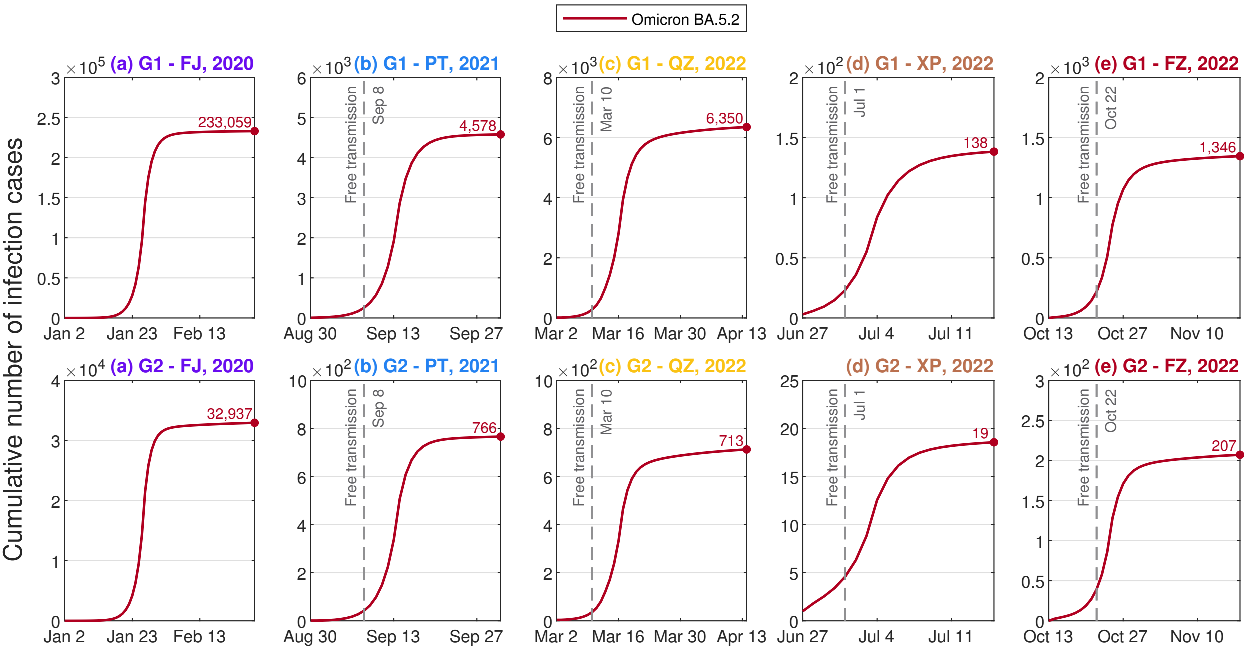

(e) The five epidemics under Omicron BA.5.2.

Supplementary Fig 2. Scale comparisons of G1 and G2. Cross-simulations of infection scales with cross-variant against surveillance data from Fujian CDC were carried out. The cumulative numbers of infection cases of G1 and G2 increased when SARS-CoV-2 variants developed with the time, in which the requirements of the medical resources showed the enhancement tendencies.

2.3. *Effective reproduction number with cross-variant* 121

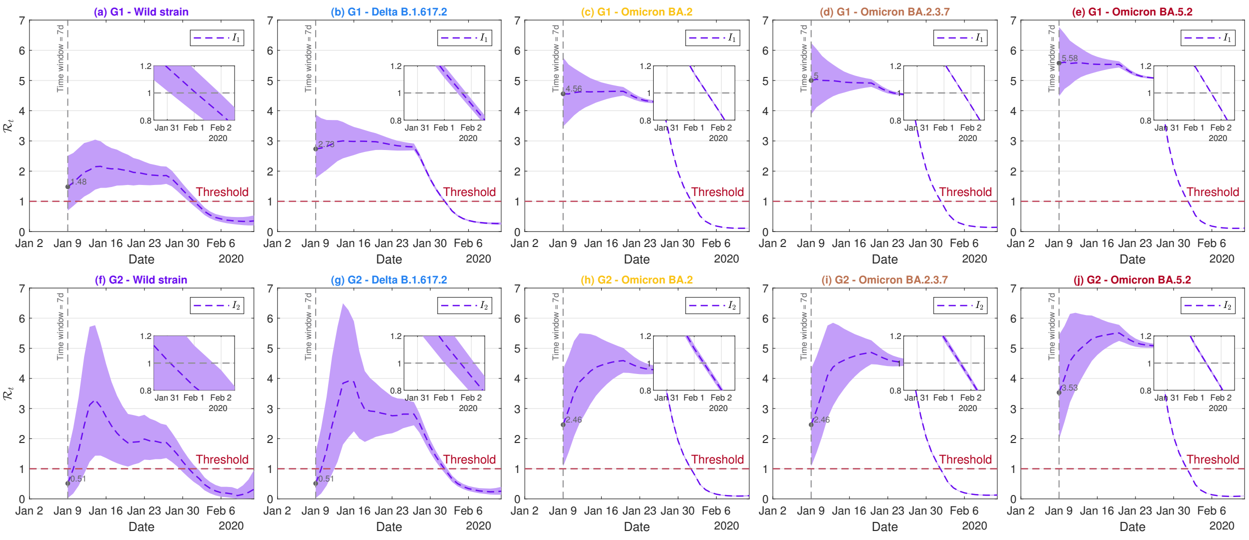

(a) Fujian epidemic under the five variants.

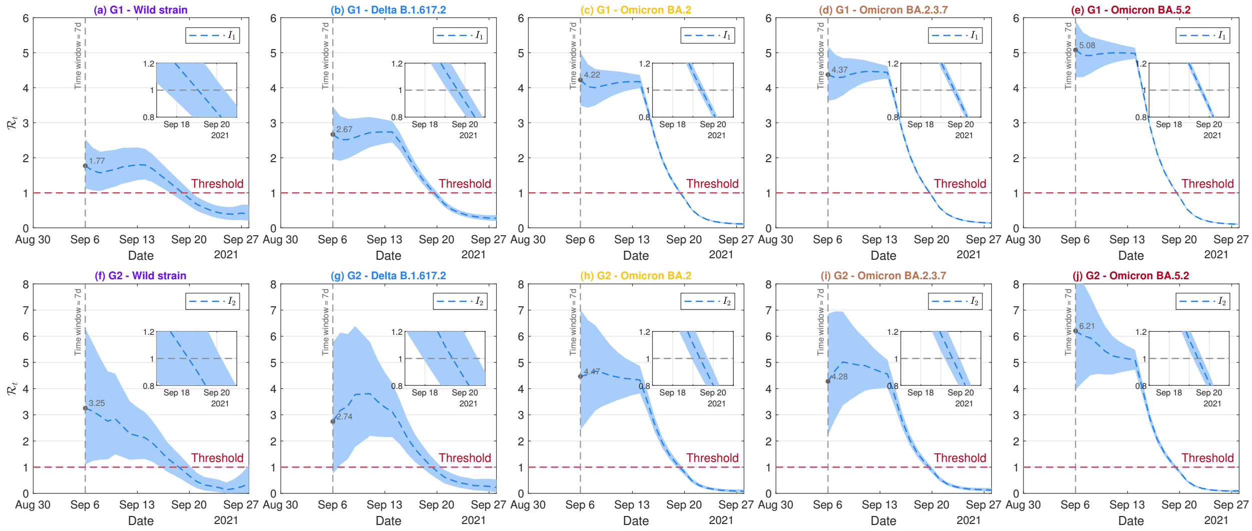

(b) Putian epidemic under the five variants.

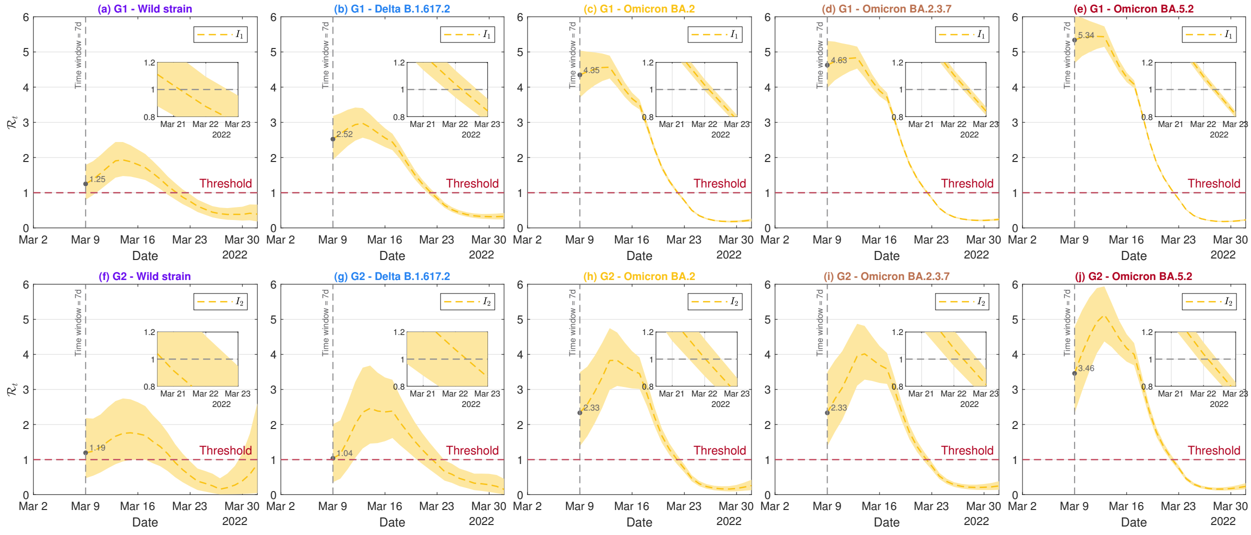

(c) Quanzhou epidemic under the five variants.

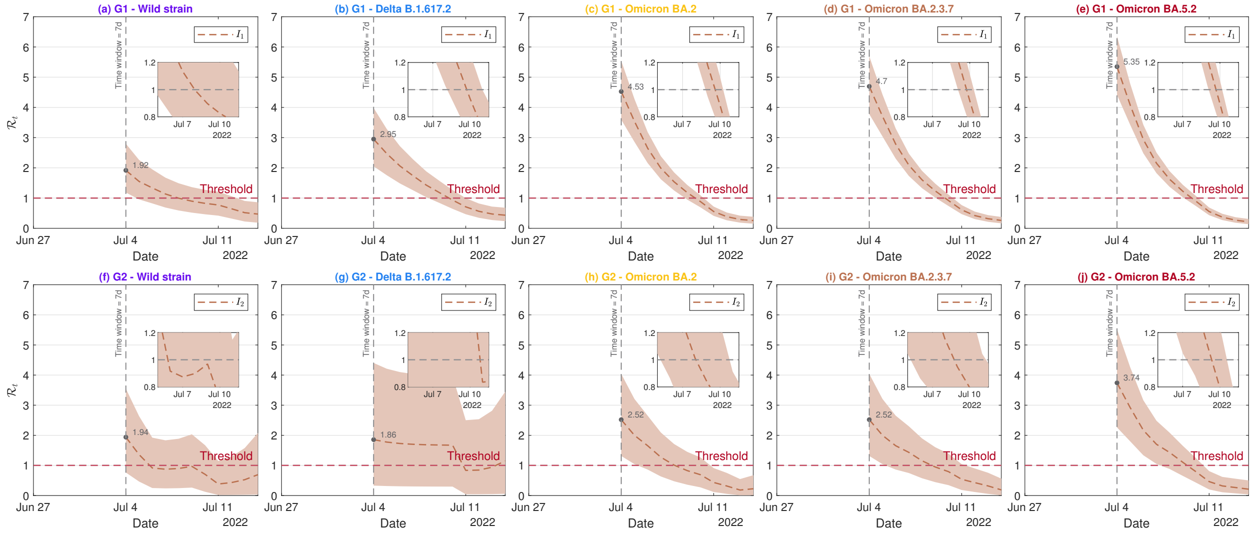

(d) Xiapu epidemic under the five variants.

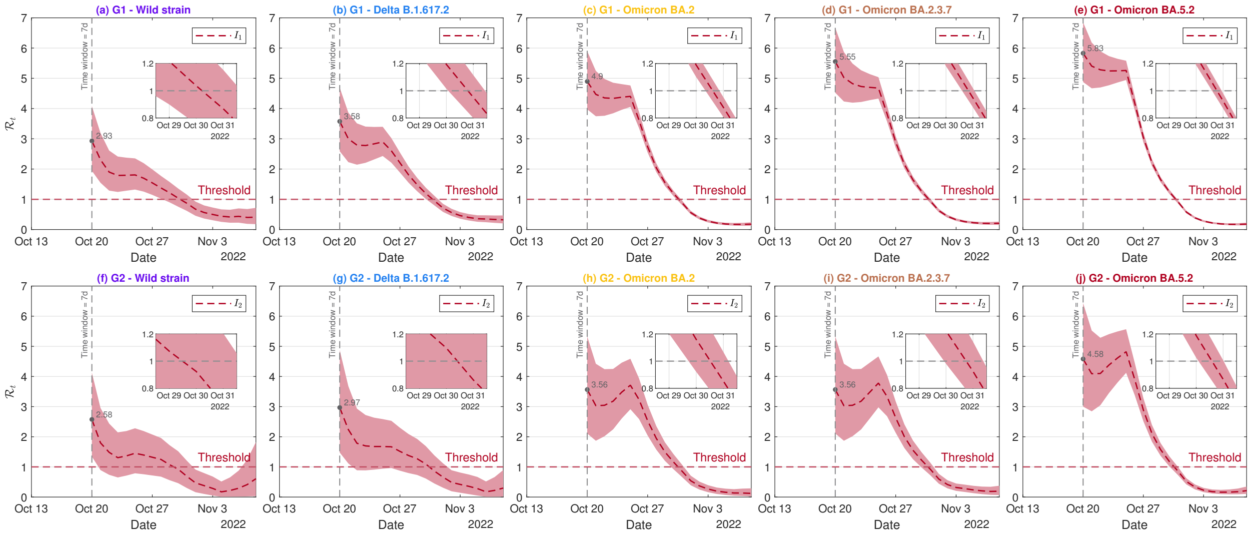

(e) Fuzhou epidemic under the five variants.

Supplementary Fig 3. Tendencies of the effective reproduction number $\mathcal{R}_t$ of the five epidemics of Fujian Province under the five variants.

2.4. Scenario investigations

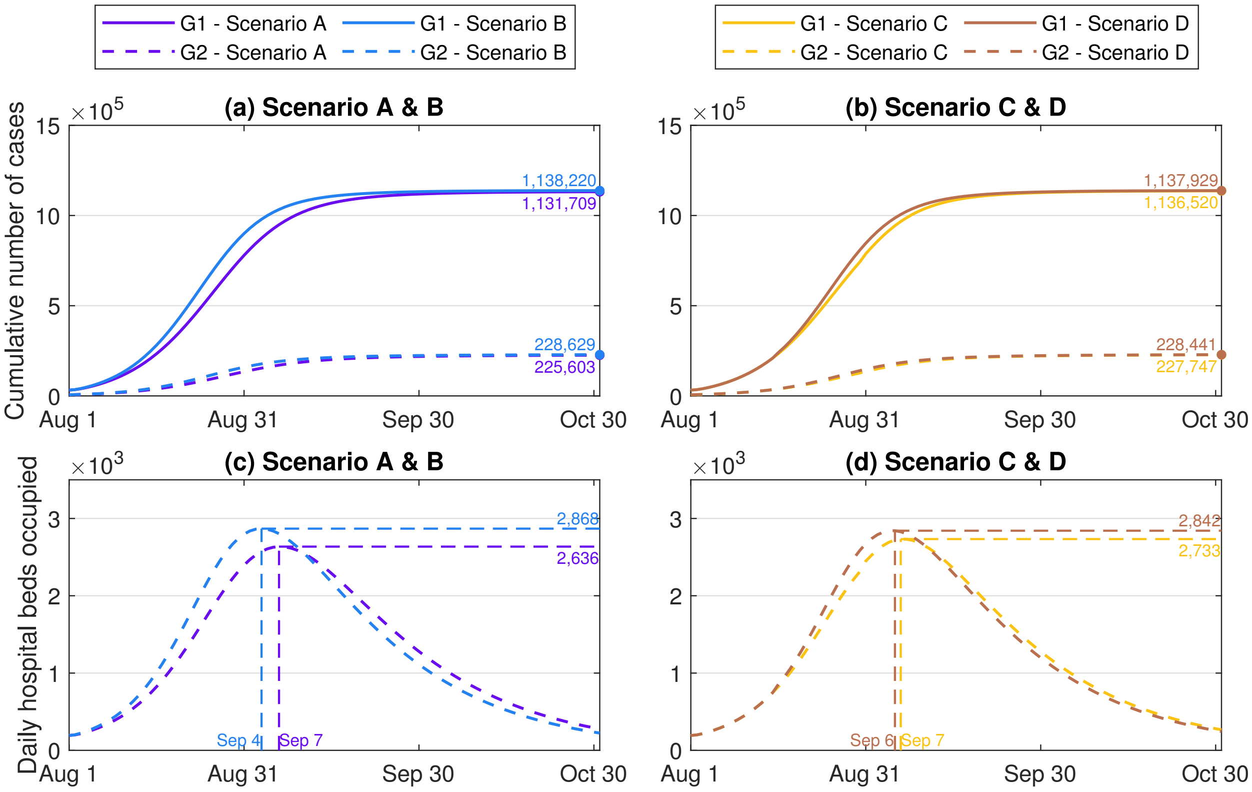

Supplementary Fig 4. Scales and peaks under four scenarios as of October 31, 2023. (a)–(b) The cumulative numbers of infection cases for G1 and G2 were derived. (c)–(d) The dates that peaks appeared and the required daily hospital beds were presented for G2.

3. Supplementary tables

Supplementary table 1. Variables and descriptions of the SVEIR model.

Variable	Description	Initials	Fujian Province, 2020		Putian City, 2021	
			Value	Source	Value	Source
S_1	No. of the susceptible in G1	$S_1(0)$	34,901,976	Computed	7,676,808	Computed
S_2	No. of the susceptible in G2	$S_2(0)$	6,638,104	Computed	1,684,014	Computed
V_1	No. of the vaccinated in G1	$V_1(0)$	0	-	664,320	[4]
V_2	No. of the vaccinated in G2	$V_2(0)$	0	-	145,728	[4]
E_1	No. of the exposed in G1	$E_1(0)$	0	Fitted	11	Fitted
E_2	No. of the exposed in G2	$E_2(0)$	0	Fitted	0	Fitted
I_1	No. of the infected in G1	$I_1(0)$	4	Fitted	5	Fitted
I_2	No. of the infected in G2	$I_2(0)$	2	Fitted	1	Fitted
R_1	No. of the recovered in G1	$R_1(0)$	0	Assumed	0	Assumed
R_2	No. of the recovered in G2	$R_2(0)$	0	Assumed	0	Assumed
s_1	Percentage of the susceptible in G1	$s_1(0)$	84.02%	Computed	75.48%	Computed
s_2	Percentage of the susceptible in G2	$s_2(0)$	15.98%	Computed	16.56%	Computed
v_1	Percentage of the vaccinated in G1	$v_1(0)$	0	Computed	6.53%	Computed
v_2	Percentage of the vaccinated in G2	$v_2(0)$	0	Computed	1.43%	Computed

Supplementary table 1. Variables and descriptions of the SVEIR model. (Cont.)

Variable	Quanzhou City, 2022		Xiapu County, 2022		Fuzhou City, 2022	
	Value	Source	Value	Source	Value	Source
S_1	7,316,981	Computed	370,941	Computed	6,498,724	Computed
S_2	1,112,718	Computed	60,385	Computed	1,308,486	Computed
V_1	364,807	[5]	40,988	[6]	402,903	[7]
V_2	55,478	[5]	6,672	[6]	81,123	[7]
E_1	0	Fitted	8	Fitted	12	Fitted
E_2	0	Fitted	2	Fitted	6	Fitted
I_1	12	Fitted	3	Fitted	0	Fitted
I_2	4	Fitted	1	Fitted	0	Fitted
R_1	0	Assumed	0	Assumed	0	Assumed
R_2	0	Assumed	0	Assumed	0	Assumed
s_1	82.68%	Computed	77.44%	Computed	78.38%	Computed
s_2	12.57%	Computed	12.61%	Computed	15.78%	Computed
v_1	4.12%	Computed	8.56%	Computed	4.86%	Computed
v_2	0.63%	Computed	1.39%	Computed	0.98%	Computed

[a] G1 meant age group who were under 60 years old; G2 meant age group who were 60 years old and over.
[b] $S_1(0) = (1 - p)N(0) - E_1(0) - I_1(0) - R_1(0) - V_1(0)$, $S_2(0) = pN(0) - E_2(0) - I_2(0) - R_2(0) - V_2(0)$.
[c] $s_1(0) = S_1(0)/N(0)$, $s_2(0) = S_2(0)/N(0)$.
[d] $v_1(0) = V_1(0)/N(0)$, $v_2(0) = V_2(0)/N(0)$.

Supplementary table 2. Parameters and descriptions of the SVEIR model.

Para.	Description	Unit[f]	Fujian Province, 2020			Putian City, 2021		
			Value	Range	Source	Value	Range	Source
ΔT	Awareness delay	d	0	0	[8, 9]	9	(6, 14)	[8, 10]
Λ	Daily recruitment rate	d^{-1}	712.33	260,000[c]	[11]	27.40	10,000[c]	[12]
N	Local population	-	41,540,086	41,540,086	[13]	10,170,887	10,170,887	[12]
g	aging rate G1[a] → G2[b]	d^{-1}	4.57×10^{-5}[e]	4×10^{-4}	[14]	4.57×10^{-5}[e]	4×10^{-4}	[14]
p	Proportion of aging population[b]	-	15.98%	15.98%	[11]	17.99%	17.99%	[12]
ν_1	Vaccination rate[a]	d^{-1}	0	0	-	3.53×10^{-3}	(0, 1)	[15]
ν_2	Vaccination rate[b]	d^{-1}	0	0	-	7.74×10^{-4}	(0, 1)	[15]
μ_1	Natural death rate[a]	d^{-1}	2.87×10^{-7}	2.87×10^{-7}	[13]	2.37×10^{-7}	2.37×10^{-7}	[12]
μ_2	Natural death rate[b]	d^{-1}	1.72×10^{-5}	1.72×10^{-7}	[13]	1.42×10^{-5}	1.42×10^{-5}	[12]
β_{11}	Infection rate by I_1[a]	$p^{-1}d^{-1}$	0.13710	(0, 0.5)	[9, 16]	0.32201	(0, 0.5)	[17, 18]
β_{12}	Infection rate by I_2[a]	$p^{-1}d^{-1}$	0.10546	(0, 0.5)	[9, 16]	0.17746	(0, 0.5)	[17, 18]
β_{21}	Infection rate by I_1[b]	$p^{-1}d^{-1}$	0.16469	(0, 0.5)	[9, 16]	0.13739	(0, 0.5)	[17, 18]
β_{22}	Infection rate by I_2[b]	$p^{-1}d^{-1}$	0.13475	(0, 0.5)	[9, 16]	0.09302	(0, 0.5)	[17, 18]
$1/\alpha_1$	Mean incubation period[a]	d	5.00	(3.53, 6.6)	[19–21]	4.40	(3.70, 5.06)	[17, 19, 22, 23]
$1/\alpha_2$	Mean incubation period[b]	d	3.00	(3.53, 6.6)	[19–21]	4.10	(3.70, 5.06)	[17, 19, 22, 23]
$1/\gamma_1$	Mean recovery period[a]	d	21.00	(13.6, 33.4)	[24, 25]	21.60	(20.6, 34.0	[17]
$1/\gamma_2$	Mean recovery period[b]	d	28.00	(13.6, 33.4)	[24, 25]	24.00	(20.6, 34.0	[17]
d_1	Death rate by virus[a]	d^{-1}	1.49×10^{-6}	0.9×10^{-2}	[16, 20, 26]	2.66×10^{-6}	0.7×10^{-2}	[27–29]
d_2	Death rate by virus[b]	d^{-1}	6.98×10^{-7}	0.9×10^{-2}	[16, 20, 26]	1.57×10^{-6}	0.7×10^{-2}	[27–30]

Supplementary table 2. Parameters and descriptions of the SVEIR model. (Cont.)

Para.	Quanzhou City, 2022			Xiapu County, 2022			Fuzhou City, 2022		
	Value	Range	Source	Value	Range	Source	Value	Range	Source
ΔT	8	(5, 14)	[8, 31]	4	(4, 14)	[8, 32]	9	(4, 14)	[8, 33]
Λ	164.38	60,000[c]	[34]	−1.48	−541[c]	[35]	76.71	28,000[c]	[36]
N	8,850,000	8,850,000	[34]	479,000	479,000	[35]	8,291,258	8,291,258	[36]
g	4.57×10^{-5}	4×10^{-4}	[14]	4.57×10^{-5}	4×10^{-4}	[14]	4.57×10^{-5}	4×10^{-4}	[14]
p	13.20%	13.20%	[34]	14.00%	14.00%	[35]	16.76%	16.76%	[36]
ν_1	1.52×10^{-3}	(0, 1)	[37, 38]	1.51×10^{-3}	(0, 1)	[39, 40]	5.83×10^{-4}	(0, 1)	[41, 42]
ν_2	2.31×10^{-4}	(0, 1)	[37, 38]	2.45×10^{-4}	(0, 1)	[39, 40]	1.17×10^{-4}	(0, 1)	[41, 42]
μ_1	3.06×10^{-7}	3.06×10^{-7}	[34]	3.53×10^{-7}	7.74×10^{-3}	[35]	2.32×10^{-7}	5.09×10^{-3}	[36]
μ_2	1.84×10^{-5}	1.84×10^{-5}	[34]	2.12×10^{-5}	2.12×10^{-5}	[35]	1.39×10^{-5}	1.39×10^{-5}	[36]
β_{11}	0.35725	(0, 0.5)	[18, 43]	0.32410	(0, 0.5)	[18, 43]	0.45123	(0, 0.5)	[18, 43]
β_{12}	0.14604	(0, 0.5)	[18, 43]	0.15443	(0, 0.5)	[18, 43]	0.26644	(0, 0.5)	[18, 43]
β_{21}	0.20100	(0, 0.5)	[18, 43]	0.19481	(0, 0.5)	[18, 43]	0.30941	(0, 0.5)	[18, 43]
β_{22}	0.15546	(0, 0.5)	[18, 43]	0.19734	(0, 0.5)	[18, 43]	0.32150	(0, 0.5)	[18, 43]
$1/\alpha_1$	2.80	(2.50, 6.60)	[19, 44, 45]	3.10	(2.50, 6.60)	[19, 44, 45]	2.75	(2.50, 6.60)	[19, 44, 45]
$1/\alpha_2$	2.60	(2.50, 6.60)	[19, 44, 45]	2.90	(2.50, 6.60)	[19, 44, 45]	2.45	(2.50, 6.60)	[19, 44, 45]
$1/\gamma_1$	18.00	(10.0, 34.0)	[46]	15.00	(10.0, 34.0)	[46]	14.00	(10.0, 34.0)	[46]
$1/\gamma_2$	21.00	(10.0, 34.0)	[46]	18.00	(10.0, 34.0)	[46]	18.00	(10.0, 34.0)	[46]
d_1	1.44×10^{-6}	0.4×10^{-2}	[28, 29]	1.93×10^{-6}	0.3×10^{-2}	[29, 47]	1.49×10^{-6}	0.25×10^{-2}	[29, 47]
d_2	7.54×10^{-7}	0.4×10^{-2}	[28–30]	6.60×10^{-7}	0.3×10^{-2}	[29, 30, 47]	6.98×10^{-7}	0.25×10^{-2}	[29, 30, 47]

[a] G1 meant age group who were under 60 years old.

[b] G2 meant age group who were 60 years old and over.

[c] Total population of Fujian Province in 2020 was 41,540,886 individuals per year, which was divided by 365 days and gave the daily recruitment rate 712.33; by the same approaches, 10,000 individuals for Putian, 60,000 individuals for Quanzhou, −540 individuals for Xiapu (− meant the loss of individuals from Xiapu County), and 28,000 individuals for Fuzhou were respectively recorded, which thus provided the corresponding values of daily recruitment rates 27.40, 164.38, −1.48, 76.71.

[d] The age distribution of the total population was assumed to be uniform for a given region. Precisely, aging rate 4.57×10^{-5} was computed by $\frac{1}{60 \times 365}$, which showed the rate/possibility that the individuals who entered G2 in one day.

[e] 1.36×10^{-5} was computed by the yearly data 4×10^{-4}, which was divided by 365 days.

[f] Unit d meant day, d^{-1} stood for per day, $p^{-1}d^{-1}$ was per person per day.

Supplementary table 3. Infectious rates of the five epidemics with the five variants in Fujian Province.

Epidemic	Phase	Period	β_{11}	β_{12}	β_{21}	β_{22}	Gradient[a]	Source
Fujian Province, 2020	1	Jan 02 – Jan 19	0.39000	0.30000	0.44000	0.36000	1.000	Fitted
	2	Jan 20 – Jan 26	0.35100	0.27000	0.39600	0.32400	0.900	Assumed
	3	Jan 27 – Mar 01	0.00039	0.00030	0.00044	0.00036	0.001	Assumed
	-	Average[b]	0.13710	0.10546	0.16469	0.13475	-	Computed
Putian City, 2021	1	Aug 30 – Sep 14	0.90000	0.49600	0.38400	0.40200	1.000	Fitted
	2	Sep 15 – Oct 01	0.00090	0.00050	0.00038	0.00040	0.001	Assumed
	-	Average	0.32201	0.17746	0.13739	0.09302	-	Computed
Quanzhou City, 2022	1	Mar 05 – Mar 16	1.36500	0.55800	0.76800	0.59400	1.000	Fitted
	2	Mar 17 – Mar 21	0.88725	0.36270	0.49920	0.38610	0.650	Fitted
	3	Mar 22 – Apr 14	0.00614	0.00251	0.00346	0.00267	0.0045	Fitted
	-	Average	0.35725	0.14604	0.20100	0.15546	-	Computed
Xiapu County, 2022	1	Jun 27 – Jul 04	1.59500	0.76000	0.77000	0.78000	1.000	Fitted
	2	Jul 05 – Jul 15	0.00638	0.00304	0.00308	0.00312	0.004	Fitted
	-	Average	0.32410	0.15443	0.19481	0.19734	-	Computed
Fuzhou City, 2022	1	Oct 16 – Oct 28	1.68000	0.99200	1.15200	1.19700	1.000	Fitted
	2	Oct 29 – Oct 30	0.03360	0.01984	0.02304	0.02394	0.020	Fitted
	3	Oct 31 – Nov 18	0.00672	0.00397	0.00461	0.00479	0.004	Fitted
	-	Average	0.45123	0.26644	0.30941	0.32150	-	Computed

[a] Gradient was calculated by values of the infectious rates in Phase 1 divided by those in Phase 2 and Phase 3, respectively.

[b] Average = $(\beta_{P_1} \times D_1 + \beta_{P_2} \times D_2 + \beta_{P_3} \times D_3)/(D_1 + D_2 + D_3)$, where β_{P_i} were the days that Phase i lasted, D_i were the infection rates during Phase i. Phase 1 included the awareness delay except for the Fujian epidemic (Fujian Province, 2020).

Supplementary table 4. Pearson correlation coefficients (PCCs) of the optimal fittings.

Epidemic	PCC of G1	PCC of G2	Epidemic	PCC of G1	PCC of G2
Fujian Province, 2020	0.9980	0.9779	Xiapu County, 2022	0.9668	0.9269
Putian City, 2021	0.9981	0.9825	Fuzhou City, 2022	0.9382	0.8796
Quanzhou City, 2022	0.9588	0.9493			

References

[1] Devaney R. An Introduction to Chaotic Dynamical Systems. New York: CRC Press; 1989.

[2] Gantmacher F. The Theory of Matrices. New York: Chelsea Publishing Company; 1964.

[3] van den Driessche P, Watmough J. Reproduction numbers and sub-threshold endemic equilibria for compartmental models of disease transmission. Math Biosci. 2002;180:29–48. doi:10.1016/S0025-5564(02)00108-6.

[4] National Health Commission of the People's Republic of China. Cumulative 82.846 million doses of SARS-CoV-2 vaccine were reported in China as of March 23 2021. 2023 [cited 9 January 2023]. Available from: `http://www.nhc.gov.cn/jkj/s7915/202103/4e9f38edab0243e6ac433a181906e527.shtml`.

[5] Quanzhou Evening News. More than 99% Quanzhou population who were 3 years old and over had been vaccinated against COVID-19. 2022 [cited 24 June 2023]. Available from: `http://qz.fjsen.com/2022-05/06/content_31026352.htm`.

[6] Xiapu Government. Implementations of vaccination plan of COVID-19 for the residents in Songcheng Street of Xiapu County. 2022 [cited 24 June 2023]. Available from: `http://www.xiapu.gov.cn/ztzl/yqfkhjjshfz/fkzc/xjzccs/202108/t20210819_1511798.htm`.

[7] Health Commission of Fujian Province. Over 10 million doses of COVID-19 have been administered in Fuzhou. 2021 [cited 9 January 2023]. Available from: `https://wjw.fujian.gov.cn/xxgk/gzdt/mtbd/202108/t20210827_5676543.htm`.

[8] Huang S, Wei F, Peng Z, Jin Z, Wang J, Xu X, et al. Assessment method of coronavirus disease 2019 outbreaks under normal prevention and control. Dis Surveill. 2020;35(8):679–686. doi:10.3784/j.issn.1003-9961.2020.08.004.

[9] Zhang W, Liu J, Zhang C, Sun Y, Huang H. Characteristics of COVID-2019 in areas epidemic from imported cases. Int J Public Health. 2020;65(6):741–746. doi:10.1007/s00038-020-01434-y.

[10] Global Times. Director Zeng Shi-Dian at Wenzhou Center for Disease Control and Prevention made the judgment on the COVID-19 tendency. 2021 [cited 24 June 2023]. Available from: `https://china.huanqiu.com/article/44lhjz9Vj6z`.

[11] Fujian Provincial Bureau of Statistics. 2021 The Seventh National Population Census Bulletin of Fujian Province (No. 2). 2021 [cited 24 June 2023]. Available from: `https://tjj.fujian.gov.cn/xxgk/tjgb/202105/t20210520_5598804.htm`.

[12] Putian Municipal Bureau of Statistics. 2021 Putian City Statistical Bulletin on National Economic and Social Development. 2022 [cited 24 June 2023]. Available from: https://www.putian.gov.cn/zwgk/tjxx/tjgb/202204/P020220415330702144569.pdf.

[13] Fujian Provincial Bureau of Statistics. 2021 Fujian Provincial Statistical Bulletin on National Economic and Social Development. 2021 [cited 24 June 2023]. Available from: https://tjj.fujian.gov.cn/xxgk/tjgb/202203/t20220308_5854870.htm.

[14] Office of the Leading Group of the State Council. Major Figures on 2020 Population Census of China. Beijing: China Statistics Press; 2021.

[15] Putian Government. About 0.95 million residents of Putian City finished the first dose, the second dose was planned to be done before June 30, 2021. 2021 [cited 24 June 2023]. Available from: https://www.putian.gov.cn/zwgk/ptdt/ptyw/202106/t20210609_1619200.htm.

[16] Fanelli D, Piazza F. Analysis and forecast of COVID-19 spreading in China, Italy and France. Chaos Solitons Fractals. 2020;134:109761. doi:10.1016/j.chaos.2020.109761.

[17] Wang Y, Chen R, Hu F, Lan Y, Yang Z, Zhan C, et al. Transmission, viral kinetics and clinical characteristics of the emergent SARS-CoV-2 Delta VOC in Guangzhou, China. eClinicalMedicine. 2021;40:101129. doi:10.1016/j.eclinm.2021.101129.

[18] Wang L, Berger NA, Kaelber DC, Davis PB, Volkow ND, Xu R. COVID infection rates, clinical outcomes, and racial/ethnic and gender disparities before and after Omicron emerged in the US. medRxiv. 2022; p. 2022.02.21.22271300. doi:10.1101/2022.02.21.22271300.

[19] Wu Y, Liu M. The incubation period of COVID-19 caused by different SARS-CoV-2 variants. Chin Gen Practice. 2022;25(11):1309. doi:10.12114/j.issn.1007-9572.2022.0078.

[20] Guan W, Ni Z, Hu Y, Liang W, Ou C, He J, et al. Clinical characteristics of coronavirus disease 2019 in China. N Engl J Med. 2020;382(18):1708–1720. doi:10.1056/NEJMoa2002032.

[21] Zhu W, Zhang M, Pan J, Yao Y, Wang W. Effects of prolonged incubation period and centralized quarantine on the COVID-19 outbreak in Shijiazhuang, China: a modeling study. BMC Med. 2021;19(1):308. doi:10.1186/s12916-021-02178-z.

[22] Park SW, Sun K, Abbott S, Sender R, Bar-On YM, Weitz JS, et al. Inferring the differences in incubation-period and generation-interval distributions of the Delta and Omicron variants of SARS-CoV-2. Proc Natl Acad Sci USA. 2023;120(22):e2221887120. doi:10.1073/pnas.2221887120.

[23] Fujian Government. Press conference on the prevention and control of COVID-19 in Fujian Province in September 29, 2021. 2021 [cited 24 June 2023]. Available from: http://www.news.cn/mrdx/2021-09/30/c_1310219319.htm.

[24] Wu S, Xue L, Legido-Quigley H, Khan M, Wu H, Peng X, et al. Understanding factors influencing the length of hospital stay among non-severe COVID-19 patients: A retrospective cohort study in a Fangcang shelter hospital. PLoS One. 2020;15(10):e0240959. doi:10.1371/journal.pone.0240959.

[25] Mizrahi B, Shilo S, Rossman H, Kalkstein N, Marcus K, Barer Y, et al. Longitudinal symptom dynamics of COVID-19 infection. Nat Commun. 2020;11(1):6208. doi:10.1038/s41467-020-20053-y.

[26] The Novel Coronavirus Pneumonia Emergency Response Epidemiology Team. Vital surveillances: The epidemiological characteristics of an outbreak of 2019 Novel Coronavirus Diseases (COVID-19) — China, 2020. China CDC Wkly. 2020;2(8):113–122. doi:10.46234/ccdcw2020.032.

[27] Ward IL, Bermingham C, Ayoubkhani D, Gethings OJ, Pouwels KB, Yates T, et al. Risk of COVID-19 related deaths for SARS-CoV-2 omicron (B.1.1.529) compared with delta (B.1.617.2): Retrospective cohort study. BMJ. 2022;378:e070695. doi:10.1136/bmj-2022-070695.

[28] Wang C, Liu B, Zhang S, Huang N, Zhao T, Lu QB, et al. Differences in incidence and fatality of COVID-19 by SARS-CoV-2 Omicron variant versus Delta variant in relation to vaccine coverage: A world-wide review. J Med Virol. 2023;95(1):e28118. doi:10.1002/jmv.28118.

[29] Tabatabai M, Juarez PD, Matthews-Juarez P, Wilus DM, Ramesh A, Alcendor DJ, et al. An analysis of COVID-19 mortality during the dominancy of Alpha, Delta, and Omicron in the USA. J Prim Care Community Health. 2023;14:21501319231170164. doi:10.1177/21501319231170164.

[30] Strasser ZH, Greifer N, Hadavand A, Murphy SN, Estiri H. Estimates of SARS-CoV-2 Omicron BA.2 subvariant severity in New England. JAMA Netw Open. 2022;5(10):e2238354. doi:10.1001/jamanetworkopen.2022.38354.

[31] Quanzhou Government. The notice of prevention and control against COVID-19 in Fengze District of Quanzhou City (No. 1). 2022 [cited 24 June 2023]. Available from: http://mzj.quanzhou.gov.cn/mzyw/tzgg/202203/t20220313_2707227.htm.

[32] Ningde Government. The 14th press conference on the prevention and control of COVID-19 in Ningde. 2022 [cited 24 August 2023]. Available from: https://www.ningde.gov.cn/ztzl/tctjyqfkhjjshfz/xwfbh/202207/t20220706_1639986.htm.

[33] Fuzhou Government. Press conference on the prevention and control of COVID-19 in Fuzhou in October 29, 2022. 2022 [cited 20 August 2023]. Available from: http://www.fuzhou.gov.cn/zgfzzt/swjw/fzwj/wjxw/gzdt_35275/202210/t20221029_4458783.htm.

[34] Quanzhou Municipal Bureau of Statistics. 2021 Quanzhou City Statistical Bulletin on National Economic and Social Development (No. 1). 2022 [cited 24 June 2023]. Available from: http://tjj.quanzhou.gov.cn/tjzl/tjgb/202204/t20220419_2718483.htm.

[35] Xiapu Municipal Bureau of Statistics. Xiapu Statistical Yearbook 2022. 2022 [cited 24 June 2023]. Available from: https://www.xiapu.gov.cn/zwgk/zfxxgkzdgz/tjxx/tjnj/202211/t20221121_1684225.htm.

[36] Fuzhou Municipal Bureau of Statistics. Fuzhou Statistical Yearbook 2022. 2022 [cited 24 June 2023]. Available from: http://tjj.fuzhou.gov.cn/zz/fztjnj/2022tjnj/indexch.htm.

[37] Fujian Government. Vaccination Status of COVID-19 in Fujian Province on March 10, 2022. 2022 [cited 24 June 2023]. Available from: http://www.fujian.gov.cn/zwgk/ztzl/yqfk/yqdt/202203/t20220311_5857438.htm.

[38] Fujian Government. Vaccination Status of COVID-19 in Fujian Province on April 14, 2022. 2022 [cited 24 June 2023]. Available from: http://www.fujian.gov.cn/zwgk/ztzl/yqfk/yqdt/202204/t20220415_5892644.htm.

[39] Ningde Government. Vaccination status on July 1, 2022. 2022 [cited 24 June 2023]. Available from: http://www.ningde.gov.cn/ztzl/tctjyqfkhjjshfz/ymjz/ymjzqk/202207/t20220703_1638768.htm.

[40] Ningde Government. Vaccination status on July 15, 2022. 2022 [cited 24 June 2023]. Available from: http://www.ningde.gov.cn/ztzl/tctjyqfkhjjshfz/ymjz/ymjzqk/202207/t20220717_1642708.htm.

[41] Fujian Government. Vaccination status of COVID-19 in Fujian Province on October 22, 2022. 2022 [cited 24 June 2023]. Available from: http://www.fujian.gov.cn/zwgk/ztzl/yqfk/yqdt/202210/t20221023_6021518.htm.

[42] Fujian Government. Vaccination status of COVID-19 in Fujian Province on November 18, 2022. 2022 [cited 24 June 2023]. Available from:

http://www.fujian.gov.cn/zwgk/ztzl/yqfk/yqdt/202211/t20221119_6057714.htm.

[43] Miyahara R, Tamura K, Kato T, Nakazaki M, Otani K, Ko YK, et al. SARS-CoV-2 variants and age-dependent infection rates among household and nonhousehold contacts. Emerg Infect Dis. 2023;29(8):1648–1650. doi:10.3201/eid2908.221582.

[44] Ogata T, Tanaka H. SARS-CoV-2 incubation period during the Omicron BA.5-Dominant period in Japan. Emerg Infect Dis. 2023;29(3):595–598. doi:10.3201/eid2903.221360.

[45] Wang K, Guo Z, Zeng T, Sun S, Lu Y, Wang J, et al. Transmission characteristics and inactivated vaccine effectiveness against transmission of SARS-CoV-2 Omicron BA.5 variants in Urumqi, China. JAMA Netw Open. 2023;6(3):e235755. doi:10.1001/jamanetworkopen.2023.5755.

[46] Wang Z, Cryar A, Lemke O, Tober-Lau P, Ludwig D, Helbig ET, et al. A multiplex protein panel assay for severity prediction and outcome prognosis in patients with COVID-19: An observational multi-cohort study. eClinicalMedicine. 2022;49:101495. doi:10.1016/j.eclinm.2022.101495.

[47] Ge J, Wang W. Vaccination games in prevention of infectious diseases with application to COVID-19. Chaos Solitons Fractals. 2022;161:112294. doi:10.1016/j.chaos.2022.112294.
